# How do rehomed laboratory beagles behave in everyday situations? Results from an observational test and a survey of new owners

**DOI:** 10.1371/journal.pone.0181303

**Published:** 2017-07-25

**Authors:** Dorothea Döring, Ophelia Nick, Alexander Bauer, Helmut Küchenhoff, Michael H. Erhard

**Affiliations:** 1 Chair of Animal Welfare, Ethology, Animal Hygiene and Husbandry, Veterinary Faculty, Department of Veterinary Sciences, LMU Munich, Munich, Germany; 2 Statistical Consulting Unit StaBLab, Department of Statistics, LMU Munich, Munich, Germany; Universidade do Porto Instituto de Biologia Molecular e Celular, PORTUGAL

## Abstract

When laboratory dogs are rehomed into private households, they experience an extreme change in their life situation. They leave their familiar, limited environment in the research facility and encounter a multitude of animate and inanimate stimuli in their new home. Although literature reports have described the experiences with rehoming as being mostly positive, scientific observations of the dogs in everyday situations have not been done. Hence, we conducted an observational test with 74 laboratory beagles 6 weeks after adoption in their new homes. This test included standardized tasks and elements; the dogs were observed during specific interactions with their new owners and during a walk. Furthermore, the owners of these 74 and of 71 additional dogs participated in standardized phone interviews 1 and 12 weeks after adoption, during which they answered questions about the dogs’ behavior in everyday situations. In the observational test, the dogs behaved mostly friendly towards humans and dogs, were tolerant during manipulations by the owner and were relaxed during the walk, even in traffic. Eighty percent (of n = 71) of the dogs walked well behaved on the leash without pulling. According to the interviews, the majority of the dogs showed desired, friendly and relaxed behavior, and the survey results reflected the bonding between dog and owner. The analysis of a possible influence of various factors (age, sex, origin, etc.) using mixed regression models confirmed the results from two previous behavior tests and interviews. Specifically, dogs that had been bred in the research facility scored significantly better than dogs that the research facility had purchased from commercial laboratory dog breeders (*p* = 0.0113). The results of this study demonstrate a successful adaptation of the rehomed beagles to their new life situation.

## Introduction

In light of the significant public interest in the fate of laboratory dogs, their rehoming into private households should be enabled [1]. In Germany, many companies and universities have been facilitating such rehoming for many years, and reports indicated mostly positive experiences with this process [2]. The German Animal Welfare Act [3] declares the killing of vertebrates “without sound reason” a punishable offense. According to the lawyers Lorz and Metzger [4], a “sound reason” for killing can exist when surplus laboratory animals cannot be placed with qualified and sensible persons. As rehoming practice in Germany shows that appropriate new owners can be found and that the dogs seem to adapt easily [2], no sound reason exists to euthanize surplus or post-experimental laboratory dogs unless they would experience pain and suffering if kept alive. From a moral standpoint, humans have an ethical obligation to provide healthy animals with appropriate living conditions.

Two options are available for the rehoming: Some facilities transfer their laboratory dogs directly to private persons. In this case, they are advised to conclude a contract with the new owner [5, 6]. However, most dogs in Germany are rehomed through specialized animal welfare organizations with long-term experience in this process. This option allows the research facilities to remain anonymous and delegate the necessary details to the organizations, whose staff members carefully select new owners, guide them through the process and conclude the contracts.

To address the placement of post-experimental or surplus laboratory dogs, the Laboratory Animal Science Association (LASA) initiated a Rehoming Guidance Working Party and organized a meeting on this topic in June 2000 [2]. During this meeting, the participants identified the need for research studies that would provide data on the rehoming of laboratory dogs [2]. The few publications that addressed rehoming until now presented experiential reports or surveys of the new dog owners [5–9], but none presented behavioral studies. Thus, we conducted a study with 145 laboratory beagles that were placed into private homes by a pharmaceutical company. In a previous publication, we described results from a behavior test that had been conducted before and 6 weeks after the placement [10]. The study also included data from two phone interviews with the new owners conducted 1 and 12 weeks after rehoming of the dogs [10]. The results indicated that the dogs adapted well to their new homes. According to their owners, they showed more relaxed and desired behavior after 12 weeks than in the first week. The main behavior problems reported by the owners were separation problems (28% of n = 125 dogs) and house soiling (39% of n = 126 dogs). The owners were very satisfied with their dogs. The majority (92% of n = 123 owners) said that they would again decide to adopt a laboratory dog. Nine dogs were returned by their owners resulting in a success rate of 94% (of n = 145). The initial behavior test in the research facility was lowly to moderately correlated with the behavior of the dogs after rehoming.

To get a broad picture of the dogs’ adaptability to everyday life and the relationship between dog and owner, we collected additional data during a home visit by observing each dog in defined situations in the home and during a walk outdoors (observational test). In addition, we analyzed further information collected in the two phone interviews, in which the new owners answered questions about their dog’s behavior in everyday situations. We further aimed to detect if factors such as age, sex, and origin of the dogs and characteristics of the new owners and households influenced the behavior of the dogs in the observational test. Finally, we were interested in the correlation between the results from this and the previous study with regard to both behavior tests (before and after rehoming) and the interviews.

## Materials and methods

### Animals and housing in the laboratory

We included 145 purpose-bred laboratory beagles from a German pharmaceutical company (Bayer AG, Leverkusen, Germany) into our study. The dogs were 65 males and 80 females from 2 months to 7.9 years of age (average age ± standard deviation of 2.2 ± 1.5 years) and had been kept, mostly singly, in 6 m^2^ indoor kennels in the research facility. At least once a day, they had had access to an outdoor run. The kennels had been equipped with a sleeping box, wooden bite sticks and dog treats. More information on the housing conditions can be found in Döring et al. [10]. The dogs were familiar with medical procedures like drawing blood, general examinations, oral applications and vaccinations.

### Rehoming

The pharmaceutical company gave the dogs to a rescue group (“Laborbeaglehilfe”) and an animal shelter (“Tierheim Wermelskirchen”), both with many years of experience in the rehoming of laboratory dogs. These two agencies selected the new owners and handled the transfer.

### Methods

**Fig 1:**
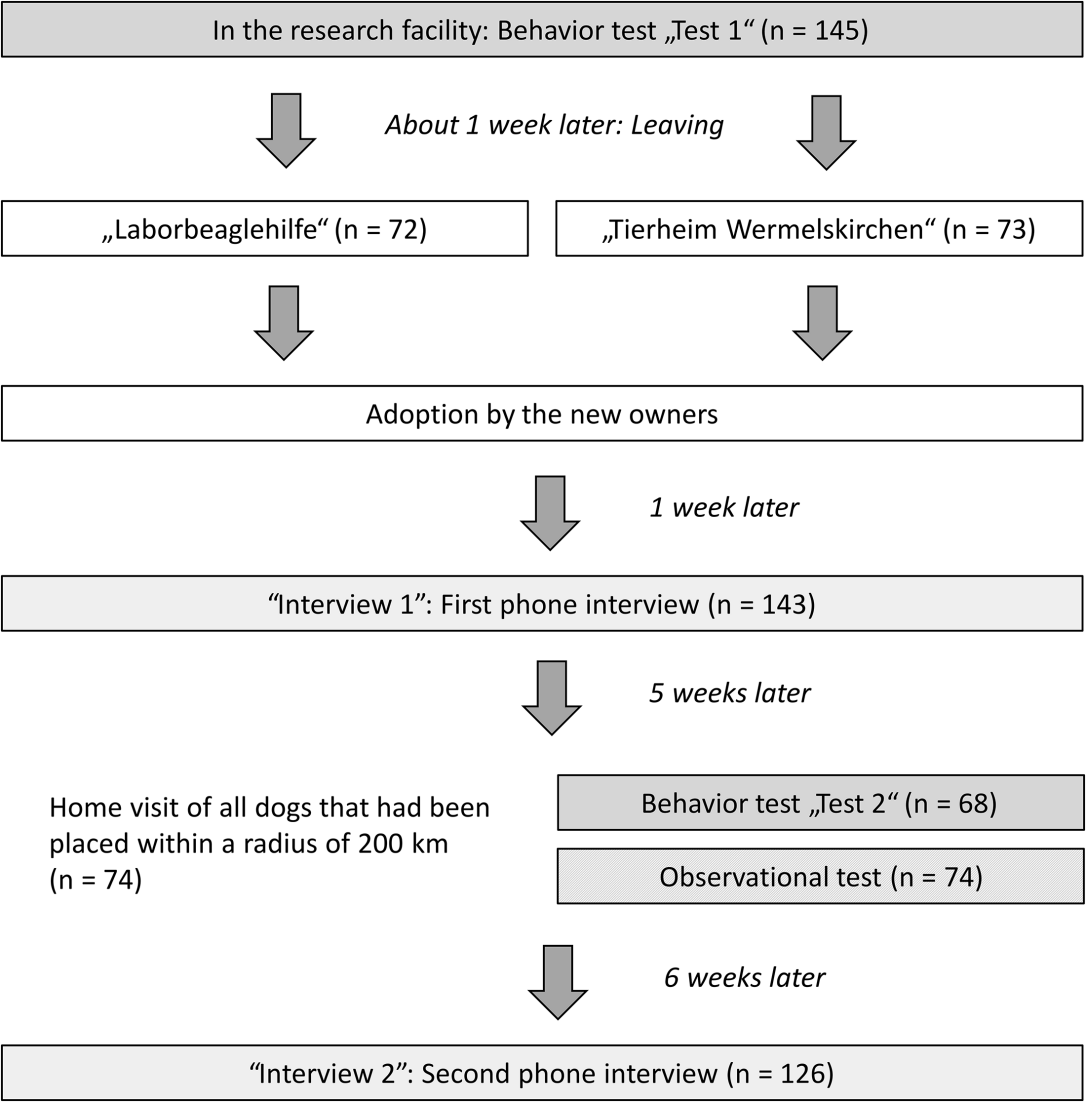
Overview of the course of events of the study.

Before the rehoming, all dogs (n = 145) were individually tested in the research facility (Test 1). This behavior test consisted of 15 test parts (description in Döring et al. [10] and in S1 Table): isolation (dog alone for 90 seconds), contact (test person enters the test arena and stands motionless for 60 seconds), luring (test person squats down and claps hands), following (test person strides once around the test arena), playing (test person rolls a rubber ball across the floor), chasing (test person pulls a shuttlecock on a string past the dog), provocation (test person grips the muzzle and holds it shut for 10 seconds), confrontation with an unknown object (test person shakes an empty plastic bag open, twists the bag and places it on the floor), confrontation with an unknown noise (test person rings a bicycle bell), examination (test person squats down and examines both of the dog’s ears, opens the dog’s mouth, lifts all of the dog’s legs one after the other and determines the heart rate with a stethoscope), placing collar and leash (test person squats and places collar and leash on the dog), leading on a leash (test person gets up and walks 2–4 steps with the dog, then stops and takes the leash and collar off the dog), covering with a thin cloth (test person slowly spreads a thin cloth over the dog including the head; this test was performed twice), and feeding (test person offers food on the palm). One and 12 weeks after rehoming, all new owners were called and surveyed in a standardized phone interview. Six weeks after placement, all dogs placed within a radius of 200 km from the research facility were visited in their new homes (n = 74). These performed the behavior test again (Test 2), followed by an observational test, in which the dogs were subjected to standardized test elements and observed without interference during interactions with their owners. The course of events of the study is given in Fig 1.

#### Interviews

Phone questionnaires with the new owners were conducted 1 week (Interview 1, n = 143) and 12 weeks after the placement (Interview 2, n = 126). In addition to the questionnaire described in Döring et al. [10] (see S1 Table), questions about the behavior in specific everyday situations were asked (Table 1, S2 Table). Seven participants in Interview 1 were foster owners, who took care of a dog until placement in the final home.

**Table 1:** Phone interviews with the new owners 1 week (Interview 1) and 12 weeks (Interview 2) after adoption. Definition of the behavior categories and percentage of dogs that showed the behavior. The description of the questions asked and the complete results are given in S2 Table.

Parameters and behavior categories	Definition	Interview 1	Interview 2
**Behavior towards the new owner**			
**Owner petting the dog**		(n = 142)	(n = 124)
enjoys	dog wags tail/ rolls on back/ closes eyes/ rubs him-/herself against owner and so forth	89%	94%
**Dog seeks contact with owner**		(n = 136)	(n = 123)
frequently	dog seeks contact more than 8 times a day	39%	52%
sometimes	dog seeks contact 4–8 times a day	32%	33%
**Whereabouts of the dog during the day**		(n = 136)	(n = 124)
follows persistently	dog persistently follows the owner, stays within 2 m of the owner all day	24%	25%
stays nearby (balanced)	dog usually stays in the same room as the owner, checks where the owner is, does spend some time in other rooms or in the garden	54%	57%
**Grooming tasks (brushing, washing) performed by owner**		(n = 128)	(n = 123)
acceptance	dog tolerates the situation	90%	93%
**Behavior towards family members**			
**Behavior towards family members (>15 years of age)**		(n = 139)	(n = 123)
friendly contact	dog approaches the family member/ wags tail/ jumps up/ has a relaxed body posture/ responds happily to petting and/or play	81%	89%
**Behavior towards children (≤15 years of age) in the family**		(n = 69)	(n = 62)
friendly contact	dog approaches the child/ wags tail/ jumps up/ has a relaxed body posture/ wants to be petted and/or wants to engage in play	68%	77%
cautious contact	dog approaches the child with signals of fear	13%	11%
**Behavior towards partner dogs (in the same household)**		(n = 61)	(n = 45)
friendly contact	dog wags tail, has relaxed body posture, plays with the other dog	72%	84%
**Behavior towards owner’s cat**		(n = 38)	(n = 29)
friendly contact	dog sniffs or looks at cat, wags tail, has relaxed body posture, respects defensive behavior of the cat by withdrawing	58%	59%
does something else	dog does not seek contact and shows no change of current behavior	26%	17%
active aggression	dog approaches the cat and barks or growls or bares teeth or snaps	3%	10%
chasing behavior	dog chases the cat	3%	14%
**Behavior towards strangers and in various situations**			
**Behavior towards unknown children (≤15 years of age)**		(n = 36)	(n = 48)
friendly contact	dog approaches the child/ wags tail/ jumps up/ has a relaxed body posture/ wants to be petted and/or wants to engage in play	56%	44%
cautious contact	dog approaches the child with signals of fear	22%	10%
fear and avoidance	dog does not approach the child, dog moves away when the child approaches him/her and shows signals of fear	11%	19%
does something else	dog does not seek contact and shows no change of current behavior	8%	15%
active aggression	dog approaches the child and barks or growls or bares teeth or snaps	3%	0%
defensive aggression	dog barks or growls or bares teeth or snaps when being approached by the child	0%	13%
**Contact with passerby**		(n = 138)	(n = 121)
friendly contact	dog walks toward the person in a speedy manner with a relaxed body posture and licks/ sniffs/ jumps up	41%	50%
cautious contact	dog hesitantly approaches the person with signals of fear, watches person/ sniffs/ licks	19%	7%
fear and avoidance	dog does not approach the person, dog moves away when the person approaches him/her and shows signals of fear	22%	17%
does something else	dog does not seek contact and shows no change of current behavior	15%	23%
active aggression	dog approaches the person and barks or growls or bares teeth or snaps	1%	1%
defensive aggression	dog barks or growls or bares teeth or snaps when being approached by the person	2%	2%
**Examination by a veterinarian**		(n = 43)	(n = 83)
acceptance	dog tolerates the situation	93%	89%
**Car ride**		(n = 124)	(n = 118)
relaxed	dog is calm with relaxed body posture and without signals of fear	75%	75%
sickness	dog salivates or heaves or vomits	22%	26%

#### Observational test

The observational test included defined tasks and standardized stimuli, but it was not as standardized as the preceding behavior test (Test 2) in order to observe the dog in everyday situations and the owner–dog interactions without interfering. Therefore, the owner was asked to call the dog in his usual way, to play with the dog as he always did, etc.

The first nine test parts were conducted in the home, whereas the following test parts were conducted outdoors, where the owner kept the dog on a leash during a walk. The test procedure is listed in Table 2 and S3 Table. The unknown test dog for confrontation outdoors was always the same dog (“Lauri”, Beauceron, 6 years, intact female). To respect the dogs’ welfare, some test parts were not conducted with dogs that panicked: The ratchet sound was omitted for 11 dogs, the hide-and-seek test for 5 dogs, and the vacuum cleaner test for 10 dogs because the owners said that, due to their dog’s fear reactions, they used the vacuum cleaner only when the dog was absent. The whole test including the walk was recorded on video.

**Table 2:** Observational test. Test parts in the order they were performed and definition of the behavior categories, behavior scores and test results (percentage of dogs that showed the behavior). The description of the test parts and the complete results are given in S3 Table. Scores were only given in test parts comparable with those of the behavior tests 1 and 2 (described in Döring et al. [10], S1 Table).

Test parts and behavior categories	Definition	Score	Results
**Contact with visitor (mimicked by the test person who rings the doorbell)**			(n = 56)
friendly contact	dog walks toward the person in a speedy manner with a relaxed body posture and licks/ sniffs/ jumps up	3	36%
cautious contact	dog hesitantly approaches the person with signals of fear, watches person/ sniffs/ licks	2	23%
fear and avoidance	dog does not approach the person; dog moves away when the person approaches him/her and shows signals of fear	0	27%
**Luring (by the owner)**			(n = 73)
comes immediately	dog comes directly to the owner without hesitation	3	60%
**Examination (by the owner)**			(n = 71)
acceptance	dog tolerates the situation	3	92%
**Playing (with the owner)**			(n = 69)
plays	dog follows the toy and/or picks up the toy with his/her mouth	-	58%
**Noise (ratchet)** **First reaction**			(n = 57)
is relaxed	dog does not flinch and does not move back	3	51%
gets startled	dog flinches	1	35%
gets frightened	dog cringes or moves back	0	14%
**Subsequent reaction**			
fear and avoidance	dog moves back and/or stays at a distance, shows signals of fear	0	16%
**First hide-and-seek (with the owner)**			(n = 62)
seeks immediately	dog seeks and finds the owner without hesitation and straightaway	-	63%
**Second hide-and-seek**			(n = 54)
seeks immediately	dog seeks and finds the owner without hesitation and straightaway	-	63%
**Object (vacuum cleaner)** **First reaction**			(n = 56)
is relaxed	dog does not flinch or move back	3	39%
gets startled	dog flinches	1	45%
gets frightened	dog cringes or moves back	0	16%
**Subsequent reaction**			
fear and avoidance	dog moves back and/or stays at a distance, shows signals of fear	0	46%
**Behavior towards partner dog**			(n = 25)
friendly contact	dog is wagging his/her tail, has relaxed body posture, plays with the other dog	-	76%
does something else	dog does not seek contact and shows no change of current behavior	-	24%
**Placing collar and leash (by the owner)**			(n = 67)
acceptance	dog tolerates the situation	3	85%
**Leash-behavior**			(n = 71)
follows along	dog follows along without the dog or the owner pulling on the leash	3	80%
**Object (garbage can)** **First reaction**			(n = 57)
is relaxed	dog does not flinch or move back	3	54%
gets startled	dog flinches	1	35%
gets frightened	dog cringes or moves back	0	11%
**Subsequent reaction**			
fear and avoidance	dog moves back and/or stays at a distance, shows signals of fear	0	28%
**A car passes**			(n = 62)
**First reaction**			
is relaxed	dog does not flinch or move back	-	92%
gets startled	dog flinches	-	8%
gets frightened	dog cringes or moves back	-	0%
**Subsequent reaction**			
fear and avoidance	dog moves back and/or stays at a distance, shows signals of fear	-	2%
**A truck passes**			(n = 34)
**First reaction**			
is relaxed	dog does not flinch or move back	-	85%
gets startled	dog flinches	-	6%
gets frightened	dog cringes or moves back	-	9%
**Subsequent reaction**			
fear and avoidance	dog moves back and/or stays at a distance, shows signals of fear	-	15%
**Passerby (random encounter)**			(n = 45)
friendly contact	dog walks toward the person in a speedy manner with a relaxed body posture and licks/ sniffs/ jumps up	-	36%
does something else	dog does not seek contact and shows no change of current behavior	-	40%
**Staircase**			(n = 52)
goes immediately	dog walks up and down the stairs without hesitation	-	90%
**Object (balloon)** **First reaction**			(n = 68)
is relaxed	dog does not flinch or move back	3	57%
gets startled	dog flinches	1	32%
gets frightened	dog cringes or moves back	0	10%
**Subsequent reaction**			
fear and avoidance	dog moves back and/or stays at a distance, shows signals of fear	0	28%
**Chasing (ball of fur is pulled past the dog)**			(n = 55)
follows	dog chases after the object	-	60%
**Unknown female dog (test dog is led past the dog)**			(n = 64)
friendly contact	dog is wagging his/her tail, has relaxed body posture, plays with the other dog	3	66%
cautious contact	dog hesitantly approaches the other dog with signals of fear	1	20%
**Obedience (owner calls and commands “sit”)**			(n = 54)
obeys immediately	dog obeys the command without hesitation	-	65%
**Feeding out of hand (of the test person)**			(n = 48)
eats out of hand	dog eats the food being offered out of the hand	-	85%

### Statistical analysis and scoring

The dogs’ behavior, which was determined from the video recordings and the responses in the phone interviews, was evaluated in a descriptive analysis. Percentages were calculated from all dogs for which data were available and “n” was given separately for each test part or parameter. Behaviors that could occur were defined a priori (behavior categories, S2 and S3 Tables). The behavior in the observational test was evaluated with scores to allow a statistical comparison with the previous behavior tests 1 (in the research facility) and 2 (before conducting the observational test; [10]) and with the phone interviews. The scoring included only the test parts that were comparable to the test parts of both previous behavior tests and to the parameters of the phone interviews described in Döring et al. [10] (see S1 Table). Behavioral reactions received a behavior score from 0 to 3, with high values indicating friendly and desired behavior and low values indicating fearful or undesired behavior. We also assigned a body language score according to Döring et al. [11], ranging from 0.5 (submissive) to 3 (relaxed/erect). In addition, a personality score was calculated as a mean of the behavior scores of the various test parts or the parameters of the interviews (S1 Table). This personality score was used to explore the influence of various factors on the behavioral reactions of the dogs (as described in Döring et al. [10]).

The open-source software R, version 3.1.2 [12], was used for statistical analyses. The level of significance was 5% (α = 0.05). To compare the results of the observational test with those of the (previous) behavior test and those of the phone interviews Kruskal's Gamma [13] was used for estimating the correlations between the ordinal variables. Kruskal's Gamma is preferable over other rank correlation measures in the case of smaller contingency tables and a relatively small sample size [14]. For variables that had more than five categories or in which most of the data were in one category, Spearman's rank correlations [15] were used because Kruskal's Gamma would be instable in such cases.

Fratkin et al. [16] found that the personality of adult dogs is more consistent than the personality of puppies. Therefore, we further calculated the correlations for dogs aged 2 years or more.

To analyze the influence of various factors on the behavior, mixed regression models [17] were estimated using the function “gam” from the “mgcv” package [18, 19]. To restrict the number of variables in the main model, only age, sex and breeder (commercial versus in-house) were chosen as main variables. The individual breeders were included as random intercept. These and other variables (rehoming organization, stay in shelter, residential area, garden, children, partner dog, experience of owners, attendance in dog classes, obedience training, frequency of rewarding, frequency of punishment) were also analyzed in an explorative approach using mixed regression models combined with Forward AIC Selection [18, 19]. With Forward AIC Selection, different regression models were compared to find the model with the best fit. The age effect was included as non-linear (penalized splines) and as linear effect, and the model with the better fit was determined with a Likelihood Ratio Test. The numbers of owners who attended dog classes, conducted obedience training, and rewarded or punished their dog differed between the 2 interviews. Therefore, the model was estimated once with the variables from Interview 1 and again with those from Interview 2.

If the owners said that the dogs would panic in certain situations (e.g., vacuum cleaner) or if dogs had shown panic behavior in preceding test parts, individual test parts were excluded for animal welfare reasons (ratchet sound for 11 dogs, vacuum cleaner for 10 dogs, hide-and-seek for 5 dogs). However, to allow inclusion of these dogs in the statistical analysis (tests for correlation and mixed regression models) they received a score of 0 for the respective test parts.

Based on information from the literature [20, 21] and our own practical experience in behavior counseling, specially selected variables were examined for correlation (Kruskal's Gamma or Spearman’s rank correlation): Behavior during isolation reported by owners was tested for correlation with contact in Test 1 (behavior test in the research facility) and with behaviors reflecting the dependence on (following persistently) and the emotional bonding with the new owner (seeking contact, behavior when being petted). Furthermore, contact in Test 1 was tested for correlation with emotional bonding with owner, and occurrence of behavior problems for correlation with inclination of owners to adopt a laboratory dog in the future. Some of the relevant data are described in detail in Döring et al. [10].

### Ethical statement

All facilities were registered according to § 11 of the German Animal Welfare Act. Our study did not include animal experiments as defined by German legislation. In Germany, animal experiments are defined in accordance with the German Animal Welfare Act as "interventions and treatments for experimental purposes, which may be associated with pain, suffering or damage". We did not have to submit an application to the authorities and we did not need permission or specific approval from them.

Prior to the adoption of a dog, the new owners had to sign a written consent form—including privacy of personal data—which they got from the welfare organization. In this form the owners agreed to participate in the study. All information from the participants was processed anonymously. The data sets did not include identifying information. We did not need to approach an ethics committee for the inclusion of human participants in the sense of the Declaration of Helsinki because we did not carry out a medical or psychological study.

## Results

### Behavior reported by owners

As shown in Table 1, the majority of the new owners said that their dogs were seeking contact with them, liked being petted and willingly allowed grooming. Most of the dogs were reported to be friendly towards other family members including children and other dogs.

According to the owners, the dogs were tolerant toward the veterinarian. During car rides, three-quarters of the dogs were relaxed, whereas almost one-quarter showed carsickness. As reported by the owners, the dogs’ behavior towards passersby and unknown children was friendly to cautious.

The comparison between Interview 1 and Interview 2 showed that the occurrence of desired behaviors increased over time. Only the behavior towards unknown children, the owner’s cat and the veterinarian worsened slightly over time according to the owners.

### Observational test of behavior

The dogs showed very tolerant behavior during examination by the owner (Table 2). About two-thirds of the dogs responded to luring and play encouragement by the owner and began seeking the owner immediately during the hide-and-seek test. The test person, when she mimicked a visitor, was greeted friendly by one-third of the dogs, whereas one-quarter of the dogs showed fear or avoidance behavior. The behavior towards a partner dog was rated friendly for three-quarters and neutral for one-quarter of the dogs. Aggressive behavior was never observed.

The majority of the dogs walked well behaved on the leash, remained relaxed when cars or trucks drove by and walked up and down stairs without problems. Most dogs showed friendly or neutral behavior towards passersby. When the unknown test dog was led by, the dogs mostly showed friendly contact behavior.

Two-thirds of the dogs chased the ball made of fur that was pulled past them. Furthermore, two-thirds of the dogs responded promptly to the commands of their owners. More dogs reacted fearfully to the vacuum cleaner than to the garbage can or the balloons (Table 2). Behavior scores were lowest during contact with the visitor (mean score points: 1.82, S4 Table) and the highest during examination by the owner (mean score points: 2.83). The mean body language scores reached 2.49 to 2.92 points reflecting a predominantly relaxed body position.

### Correlation of observational test with behavior tests (1 and 2) and behavior reported by owners (Interviews 1 and 2)

The behavior scores of the observational test were correlated for 8 out of 12 parameters with those of Test 1 and for 10 out of 12 parameters with those of Test 2 (Table 3). Highest correlations were found for contact (to visitors). Correlations also existed with regard to body language. Scores of the observational test were also correlated with those of both interviews (Interview 1: 7 out of 13 parameters; Interview 2: 7 out of 13 parameters). Most correlations were low to moderate, reflecting a small to substantial relationship [22], but with Interview 2, there were high correlations regarding contact (to visitors), luring (by the owner) and the subsequent reaction to noises.

**Table 3:** Correlation of behavior scores between observational test (conducted 6 weeks after adoption) and behavior tests (Test 1 and Test 2, conducted before and 6 weeks after adoption, respectively) and between observational test and phone interviews (Interview 1 and Interview 2, conducted 1 and 12 weeks after adoption, respectively) and correlation of body language scores between observational test and both behavior tests. The scores of Test 1, Test 2, Interview 1 and Interview 2 are described in Döring et al. [10].

Correlation with		Test 1	Test 2	Interview 1	Interview 2	Test 1	Test 2
Test situation / parameter		Behavior score				Body language score	
**Contact**	n	55	54	53	49	55	54
*Kruskal’s Gamma*	γ	0.4433	0.7030	0.7870	0.7785	0.2142	0.6222
**Luring**	n	72	72	72	65	71	72
*Kruskal’s Gamma*	γ	0.3093	0.6875	0.4213	0.7176	0.2700	0.4223
**Playing** ^a)^	n	68	61	67	63	67	61
*Spearman’s rank correlations*	r	0.0825	0.4304	0.2373	0.3867	0.1073	0.1565
**Chasing** ^a)^	n	54	51	53	49	53	51
*Spearman’s rank correlations*	r	0.1976	0.2339	0.0135	−0.1913	0.1880	0.3546
**Object, 1st reaction** ^b)^	n	70	70	70	64	68	66
*Spearman’s rank correlations*	r	0.2961	0.3931	0.0951	0.2357	0.1499	0.3125
**Object, subsequent reaction** ^b)^	n	71	70	69	61	-	-
*Kruskal’s Gamma*	γ	0.6979	0.4716	0.3264	0.7269	-	-
**Noise, 1st reaction**	n	54	57	56	54	54	57
*Spearman’s rank correlations*	r	0.1538	0.2279	0.0806	0.1860	0.0067	0.2545
**Noise, subsequent reaction**	n	54	57	54	54	-	-
*Spearman’s rank correlations*	r	0.4382	0.2723	0.1087	−0.0466	-	-
**Examination** ^c)^	n	70	70	70	64	69	69
*Spearman’s rank correlations*	r	0.0040	0.0352	0.1298	−0.0366	0.0918	0.0639
**Placing collar**	n	66	66	66	60	65	65
*Spearman’s rank correlations*	r	−0.2298	−0.1281	0.0193	0.1825	−0.0986	0.4028
**Leash-behavior**	n	61	67	69	63	59	67
*Kruskal’s Gamma*	γ	-0.3898	0.4366	0.2203	0.6532	0.2294	0.5807
**Feeding** ^a)^	n	47	38	46	44	46	37
*Spearman’s rank correlations*	r	0.2039	0.4175	0.4869	−0.0949	0.0406	0.4943
**Other dogs**	n	-	-	59	57	-	-
*Kruskal’s Gamma*	γ	-	-	0.4042	0.6401	-	-

^a)^ Behavior was not scored, but presence/absence of behavior was determined (e.g., playing: Does dog play in Test 1 and Test 2, yes or no?).

^b)^ For the observational test, we calculated the mean from three object test parts: vacuum cleaner, garbage can and balloon.

^c)^ For Tests 1 and 2, mean values were calculated from 4 examinations: ears, mouth, legs and auscultation.

Colors:

White: no correlation (<0.2).

Light gray: low correlation (0.2 to <0.4).

Dark gray: moderate correlation (0.4 to <0.7).

Blue: High correlation (≥0.7).

When only the dogs aged 2 years or more were considered (S5 Table), we found a high correlation for feeding between observational test and Test 2 (r = 0.845). The test person conducted the two feeding situations on the same day, first in Test 2, then in the observational test. Furthermore, the observational test had high correlations with both tests and interviews regarding contact (to visitors).

### Correlation of specially selected variables

We found no or low correlations between the specially selected variables, which had been presumed to be related (Table 4). An exception was the behavior during separation reported by the owners, which was correlated with the owners’ subjective assessment of a separation problem; this correlation was very high in Interview 1 (γ = 0.993) and Interview 2 (γ = 0.951), reflecting a very dependable relationship [22]. According to the owners, 14% (19 of n = 138) of the dogs showed separation problems 1 week after adoption and 28% (35 of n = 125) 12 weeks after adoption [10]. Behaviors reflecting owner dependence (owner reports that the dog follows him or her persistently) had low to moderate correlation with the reported behavior during isolation. The owners’ inclination whether or not to adopt a laboratory dog again was lowly correlated with their perception of an annoying behavior of their actual dog.

**Table 4:** Correlation of specially selected variables that had been suggested to be related.

Variable 1	Variable 2	n	r or γ	Method
Behavior during isolation, Interview 1 ^a)^	Separation problems, Interview 1 ^b)^	114	−0.9929	*Kruskal’s Gamma*
Behavior during isolation, Interview 2 ^a)^	Separation problems, Interview 2 ^b)^	123	−0.9513	*Kruskal’s Gamma*
Behavior during isolation, Interview 1 ^a)^	Dog persistently follows owner, Interview 1 ^c)^	107	−0.5440	*Kruskal’s Gamma*
Behavior during isolation, Interview 2 ^a)^	Dog persistently follows owner, Interview 2 ^c)^	122	−0.3143	*Kruskal’s Gamma*
Social contact in Test 1 ^d)^	Emotional bonding with owner, Interview 1 ^e)^	138	0.1437	*Spearman’s rank*
Social contact in Test 1 ^d)^	Emotional bonding with owner, Interview 2 ^e)^	120	0.1048	*Spearman’s rank*
Social contact in Test 1 ^c^^d)^	Behavior during isolation, Interview 1 ^a)^	112	−0.0424	*Spearman’s rank*
Social contact in Test 1 ^c^^d)^	Behavior during isolation, Interview 2 ^a)^	119	0.0047	*Spearman’s rank*
Emotional bonding with owner, Interview 1 ^e)^	Behavior during isolation, Interview 1 ^a)^	113	0.0352	*Kruskal’s Gamma*
Emotional bonding with owner, Interview 2 ^e)^	Behavior during isolation, Interview 2 ^a)^	123	0.0285	*Kruskal’s Gamma*
Adopt a laboratory dog again? Interview 1 ^f)^	Behavior perceived as annoying, Interview 1^g)^	132	−0.1412	*Spearman’s rank*
Adopt a laboratory dog again? Interview 2 ^f)^	Behavior perceived as annoying, Interview 2 ^g)^	120	−0.2314	*Spearman’s rank*
Adopt a laboratory dog again? Interview 1 ^f)^	Aggressive behavior, Interview 1 ^h)^	132	−0.1159	*Spearman’s rank*.
Adopt a laboratory dog again? Interview 2 ^f)^	Aggressive behavior, Interview 2 ^h)^	120	−0.1822	*Spearman’s rank*.

^a)^ Behavior during isolation (dog left alone at home) reported by owners; calm: dog does not bark, howl or whine and does not destroy anything, score 3; unsettled: dog is unsettled and moves around, dog barks, howls or whines up to 3 minutes, score 1; separation problems: dog barks, howls or whines more than 3 minutes and/or destroys objects in the home, score 0.

^b)^ The owners were asked if they noticed separation problems (defined as dog barks, howls, whines or destroys objects when being alone in the home). No scoring, answer yes = 1 or no = 0.

^c)^ According to Table 1, parameter “Whereabouts of the dog during the day”, behavior category “follows persistently”. No scoring, answer yes = 1 or no = 0.

^d)^ Social contact: mean score of the behavior test parts contact, luring and following (description of these test parts in S1 Table, data in Döring et al. [10]).

^e)^ Emotional bonding with owner, behavior reported by owners: mean score of “Dog seeks contact with owner” (frequently = 3, sometimes = 2, rarely = 1, not at all = 0) and “Owner petting the dog” (enjoys = 3, acceptance or slight withdrawal = 2, moves away or aggression = 0, definitions according to Table 1).

^f)^ The owners were asked if they would again decide to adopt a laboratory dog. No scoring, answer yes = 1 or no = 0, data in Döring et al. [10].

^g)^ The owner was asked if the dog showed behavior perceived as annoying. No scoring, answer yes = 1 or no = 0, data in Döring et al. [10]. Owners who said “I do not know” were not included.

^h)^ The owners were asked if the dog showed threatening behavior (barking, growling or baring teeth) and/or snapping or biting. No scoring, answer yes = 1 or no = 0, data in Döring et al. [10].

Colors:

White: no correlation (<0.2).

Light gray: low correlation (0.2 to <0.4).

Moderate correlation (0.4 to <0.7) did not occur.

Blue: high correlation (≥0.7).

### Influencing factors

Of the variables examined in the main model (age, sex and breeder), a significant difference (*p* = 0.0113) existed regarding the variable breeder (S6 Table): Dogs purchased from commercial breeders scored on average by −0.7757 score points worse than dogs bred in the research facility. Although no significant sex difference existed, male dogs scored slightly (on average −0.1495 score points) worse than female dogs.

In the explorative mixed model using Forward AIC Selection, we detected as positive factors (with higher personality scores) the presence of a garden, obedience training, frequent rewarding, frequent punishment (i.e., mostly verbal punishment with words like “Aus”, “Nein” and “Pfui”, which mean “Leave it”, “No” and “Drop it”, respectively) and partner dog with estimates >0.3. Attendance in dog classes was identified as a negative factor (S6 Table): Dogs that attended dog classes scored on average by −0.4368 score points worse than dogs that did not attend dog classes.

**Fig 2:**
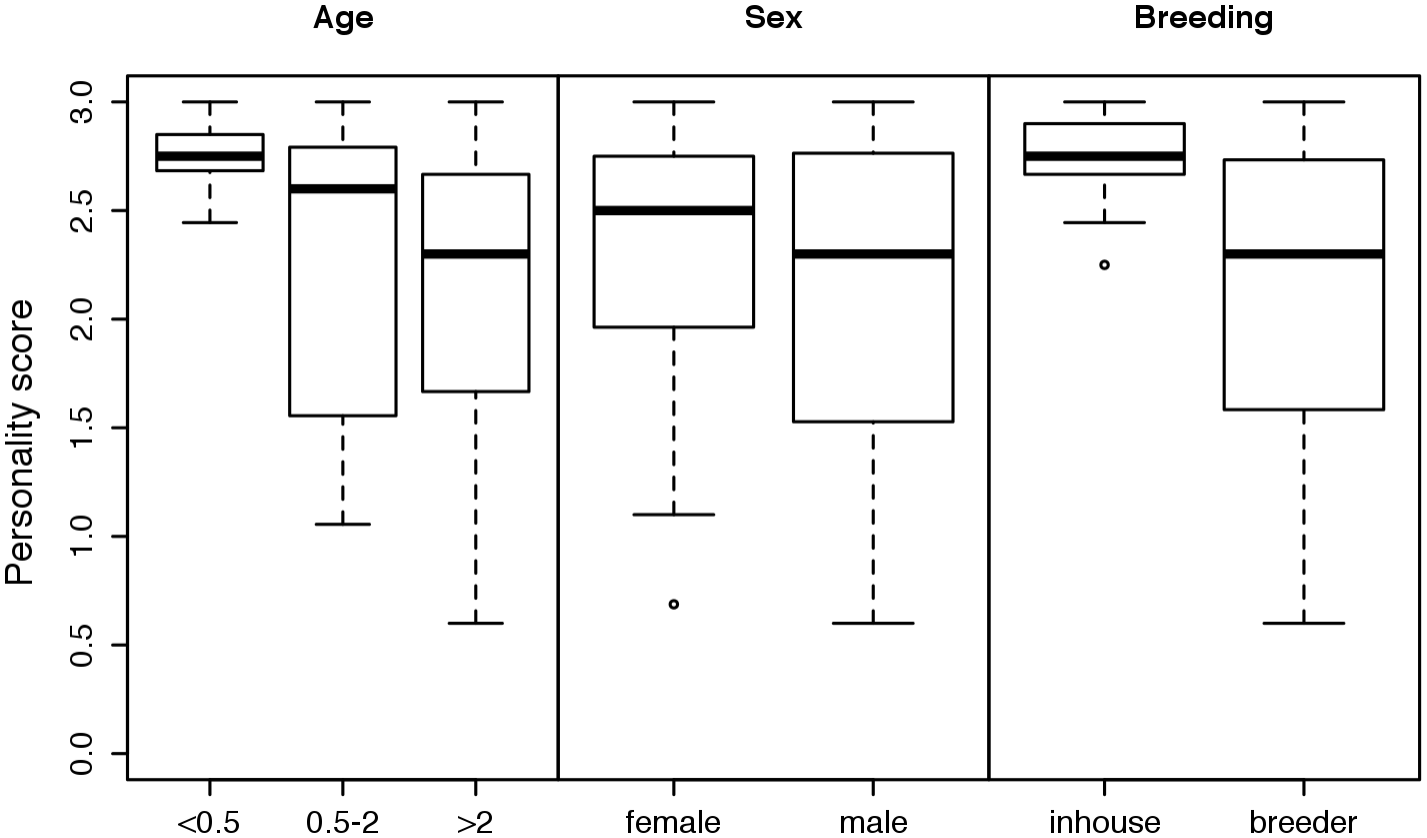
Comparison of the personality scores (mean of the scores of Table 2) according to age (in years), sex and breeder (bred in the research facility = “in-house” versus commercial breeder for laboratory dogs = “breeder”). High scores indicate relaxed/desired behaviors.

**Fig 3:**
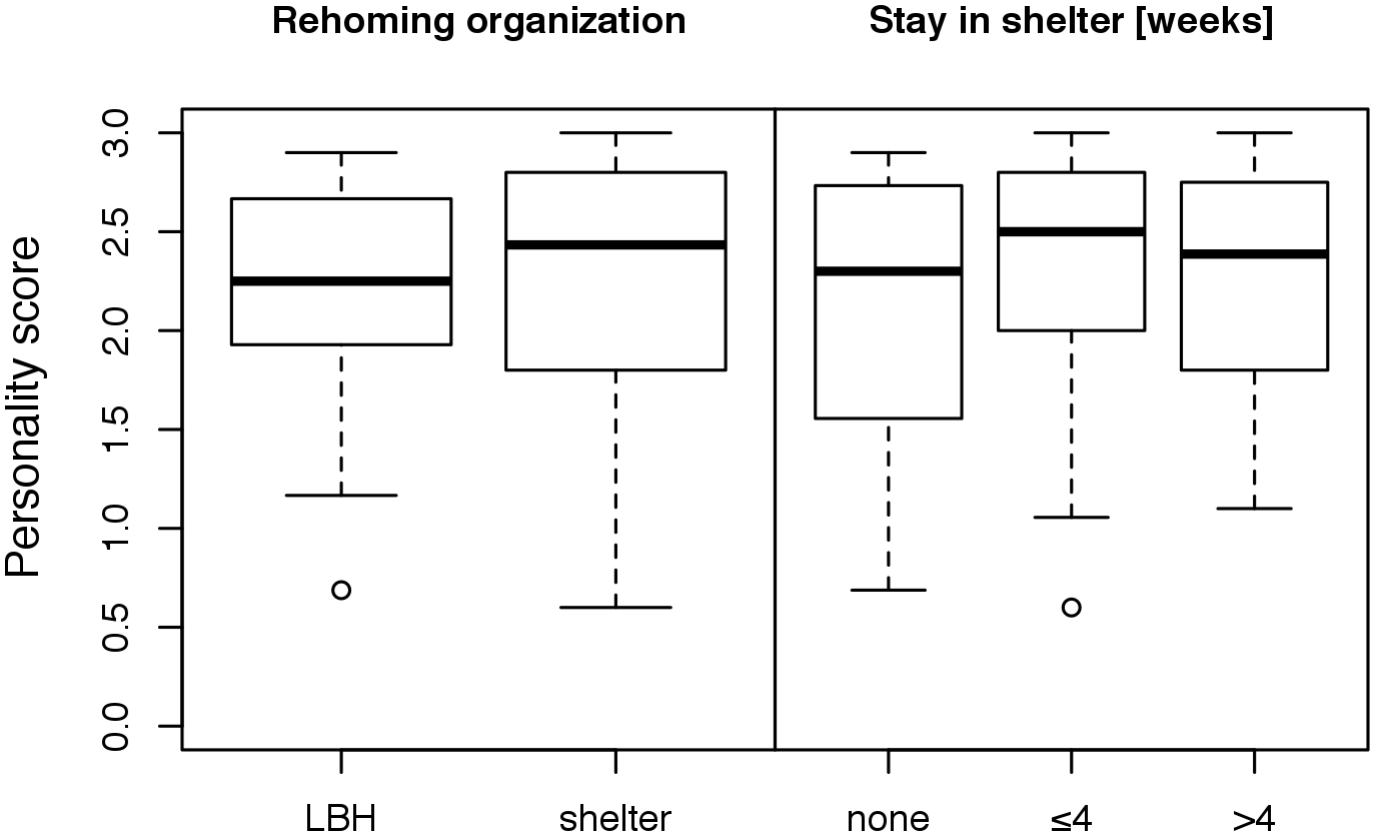
Comparison of the personality scores (mean of the scores of Table 2) according to rehoming organization (“LBH” = Laborbeaglehilfe; “shelter” = shelter Wermelskirchen) and time spent in the shelter Wermelskirchen until adoption (in weeks). High scores indicate relaxed/desired behaviors.

**Fig 4:**
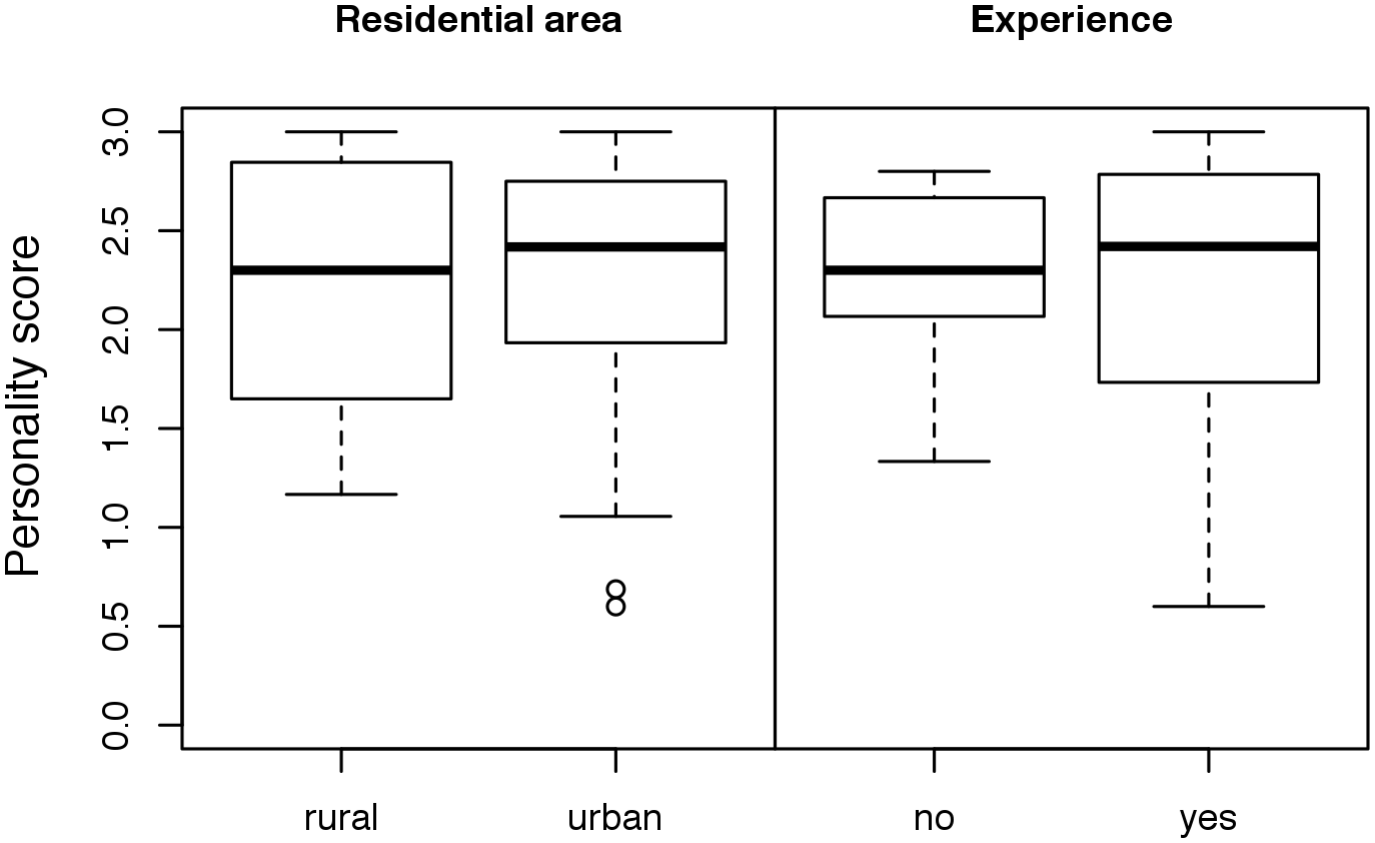
Comparison of the personality scores (mean of the scores of Table 2) according to residential area (“rural” versus “urban” = city and suburb) and dog experience of the new owner (no experience = owner had no dog before). High scores indicate relaxed/desired behaviors.

**Fig 5:**
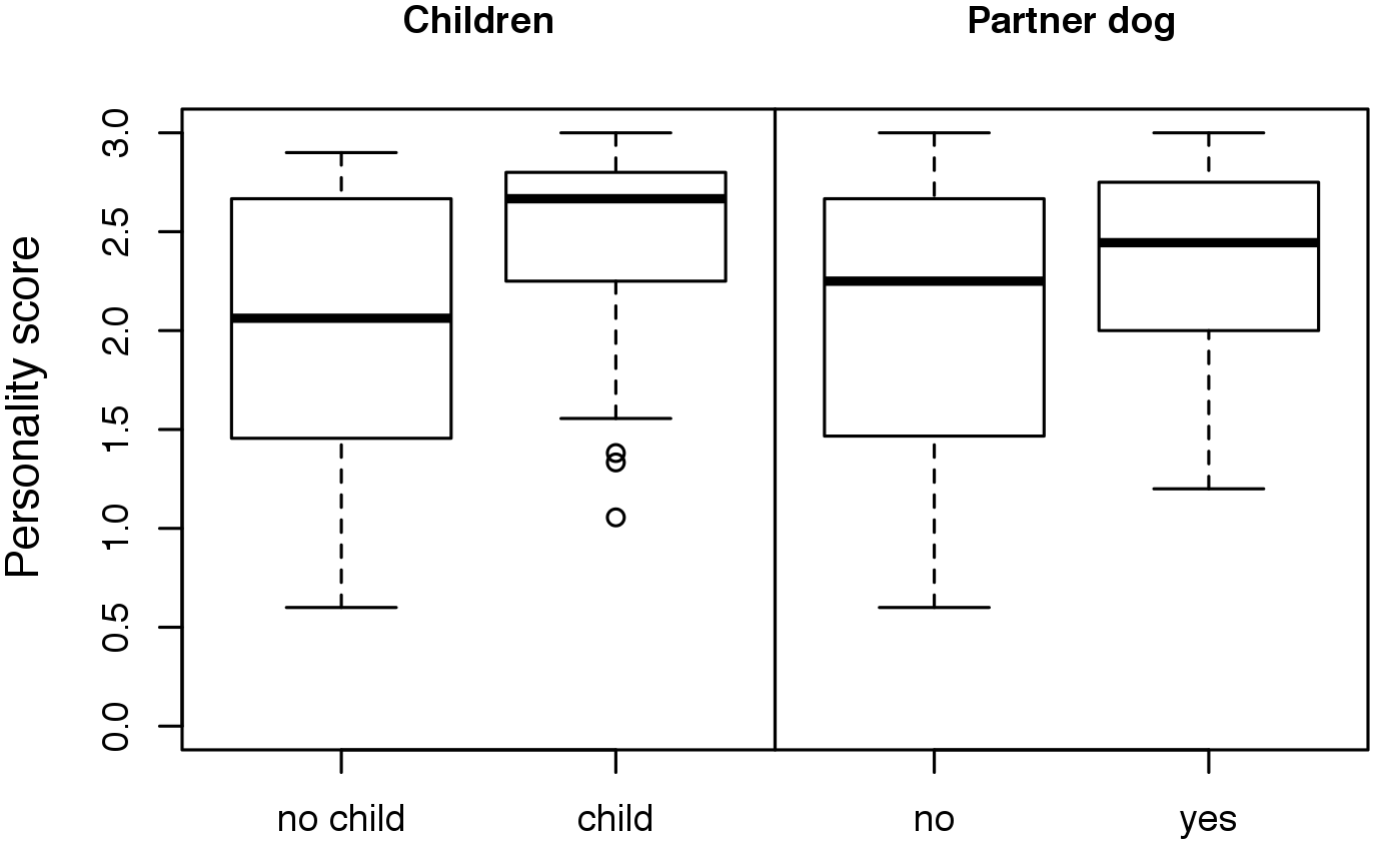
Comparison of the personality scores (mean of the scores of Table 2) according to family situation of the new owner, i.e., according to the presence/absence of a child ≤15 years old (grandchild not included) and of another dog in the household. High scores indicate relaxed/desired behaviors.

Puppies under 6 months of age, female dogs and in-house-bred dogs received higher personality scores in the observational test than older dogs, male dogs and purchased dogs, respectively (Fig 2, S7 Table). The overall low personality scores of dogs from commercial breeders were caused mainly by the low scores of dogs purchased from two German breeders (S7 Table). The dogs rehomed by the shelter Wermelskirchen scored slightly higher than those rehomed by the rescue group Laborbeaglehilfe (Fig 3); however, it should be noted that Wermelskirchen handled dogs that had scored higher in Test 1 (conducted in the research facility before the rehoming) compared with the dogs handled by Laborbeaglehilfe (see Döring et al. [10]). With regard to the variables residential area and experience of the owners, we found no unambiguous differences between the analyzed factors (Fig 4). The dogs placed in families with children received higher personality scores than those placed in households without children (Fig 5). With regard to the presence or absence of a partner dog, the distribution of personality scores in the observational test was similar to that in Test 1 before adoption [10]. Thus, the presence of a partner dog did not influence the personality score (Fig 5).

## Discussion

In the observational test, the dogs showed mostly relaxed and desired behaviors. We see these results as very positive because they demonstrate a high adaptive capacity of the rehomed laboratory dogs. Results from the interviews with the new owners also reflected the dogs’ adaptation to everyday life and their emotional bonding with their owners (more dogs that enjoyed petting and that sought contact with the owners were reported in Interview 2 than in Interview 1). Furthermore, this bonding may explain why the dogs were seeking less contact with the test person in the observational test in the new home than in the behavior test conducted in the research facility before rehoming. Other explanations may be that the dogs in the research facility were under-stimulated and therefore more interested in contact with humans or that the test person was not interesting to the dogs when she mimicked a visitor in the observational test because she was no longer a stranger to the dogs and had been gone for only a short moment.

We developed a new observational test to analyze the dogs’ everyday behavior. It included elements of the observational tests according to Jones and Gosling [23]. Our observational test proved to be well suited to evaluate the dogs’ behavior in everyday situations. It showed many correlations with the preceding behavior tests and with the statements made by the owners in the interviews. As typical for such studies, the correlations were mostly low to moderate, but many of them were higher than those found between the behavior tests and the interviews [10]. This finding indicates that, for behavioral evaluations, it is useful to observe dogs in everyday situations with their owners in addition to a repetition of the standardized testing that had initially been conducted in the research facility. Bennett et al. [24] also recommended that the evaluation of a dog should be based on more than one assessment and include as much information as possible. The high correlations with Interview 2 indicate that the behaviors observed in the observational test were consistent with the respective behaviors reported by the owners 6 weeks later.

As separation anxiety was previously associated with overattachment [20, 21], we tested the relation between the reported behavior during isolation and the occurrence of the behaviors “persistently following” and “emotional bonding” with the new owner. We found a moderate correlation for “persistently following” reflecting a substantial relationship [22] regarding this behavior. It is possible that the number of dogs with separation problems (35 out of 125 dogs after 12 weeks) was too small to detect further relationships. On the other hand, Parthasarathy and Crowell-Davis [25] did not find a connection between separation anxiety and overattachment to the owner.

To find possible relations between the social contact behavior in Test 1 in the research facility and the later occurrence of “emotional bonding” with the new owner, correlations were calculated. The test part contact in Test 1 failed to predict emotional bonding with the new owner, but it was correlated with behavior towards visitors in the new home (Table 3; [10]). In line with Döring et al.’s [10] and similar to Svartberg’s [26] results, these correlation results showed the greatest consistency in our study. This finding implies that emotional bonding with the new owner is not related to the behavior towards visitors.

After 12 weeks, 92% of 123 dog owners stated that, if facing a future decision, they would adopt a laboratory dog again [10]. This statement was only lowly correlated with the occurrence of behavior problems (Table 4), showing that the new owners were very patient and understanding. The high level of satisfaction with the adopted animal was also reflected in other surveys on the rehoming of laboratory dogs [5, 9] and cats [27].

The statistical analysis of factors that may affect the behavior yielded results similar to those obtained from the analyses of the behavior tests and the interviews [10]. The dogs bred in the research facility scored significantly higher (i.e., better) than those purchased from a commercial breeder. As found for the scores in the behavior tests and interviews [10], the dogs from two German breeders were responsible for these low scores. The dogs purchased from the US breeders received relatively high scores. Obedience training and frequent rewarding had positive effects. In contrast, attendance in dog training classes was not a positive factor in our study, whereas Diesel et al. [28] found for adopted shelter dogs that attending dog training classes significantly decreased the chance that the adoption failed. Bennett and Rohlf [29] and Kutsumi et al. [30] in their studies on companion dogs also found positive effects of attending dog training classes, especially for puppies. We argue here that it may be more important to conduct obedience training than to attend dog training classes. Furthermore, the negative effects of attendance in dog training classes seen in our study could indicate that the owners of dogs with problematic behavior may have visited dog training classes to seek professional help in addressing an existing problem. Frequent punishment had a positive effect in the explorative model. However, almost none of the new owners applied physical punishment, and punishment mostly entailed verbal interruptions of undesired behaviors. Hence, this result may be better explained as a positive effect of communication than a positive effect of punishment.

The adopted beagles showed very friendly behavior towards family members, children and partner dogs in the household. This friendly, non-aggressive disposition is typical for laboratory beagles [14, 31]. Dogs in households with children scored higher in the observational test than those in households without children, confirming the positive effect of the presence of children in the new home detected by Döring et al. [10]. Nonetheless, safety rules for the interaction between dog and child must be in place for bite prevention, regardless of the friendly and obedient temper of the former laboratory beagles. The child must not corner or disturb the dog during resting or feeding. Furthermore, child and dog must never be left unattended with each other but always be supervised by an adult [32–34].

Although our results showed that most dogs adapted well to their new lives and reacted relaxed in everyday situations, there was a considerable proportion of dogs that reacted anxiously in some situations of the observational test, e.g., when being confronted with the vacuum cleaner in operation and when a garbage can was noisily pulled past the dog. Furthermore, several dogs were not subjected to certain test parts because the owners said that the dogs would panic in such situations (e.g., vacuum cleaner) or because the dogs had panicked in previous test parts. Fearfulness has also frequently been observed in rehomed shelter dogs: In a study by Wells and Hepper [35], 53.4% of shelter dogs (n = 556) displayed fearful behavior within the first month after adoption, and Marston et al. [36] reported “generalized fear” in 32.3% of the tested dogs (n = 62), with 14.5% being fearful “most of the time or always”. Frequent fearful behavior was also reported by new owners of greyhounds, with fear in unfamiliar situations occurring most often (41.4%, [37]). Fearful behavior is common in dogs: Blackwell et al. [38] found that 49% of 383 interviewed dog owners reported behavioral signs typical of fear and 25% of 4,280 interviewed dog owners perceived their dogs as being “fearful”. Martínez et al. [39], who interviewed 232 dog owners in a veterinary clinic, estimated a prevalence of 51.7% for noise phobia and 17.7% for fear of people. Compared with these data from the literature, the laboratory dogs did not perform worse. Nevertheless, for animal welfare reasons, everything should be done to prevent fear and other behavioral problems. Therefore, a careful selection and education of the new owners is highly recommended [2]. Although no studies exist that tested these recommendations for laboratory beagles, Diesel et al. [28, 40] confirmed their usefulness based on their experience with shelter dogs. Gazzano et al. [41] showed that a 1-hour counseling of puppy owners reduced the occurrence of subsequent behavior problems. Herron et al. [42] counseled new owners of rehomed shelter dogs in a 5-minute pre-adoption session on housetraining, which reduced the subsequent need for verbal punishment and the perceived problem of elimination in the home. However, the 5-minute counseling was obviously too short to have an effect on the actual occurrence of the problem. This limitation was also shown when new owners of shelter dogs received a 5-minute pre-adoption counseling on separation anxiety [43]. For the prevention of complex behavior problems such as separation anxiety, a thorough counseling is necessary. Furthermore, the owners should be advised to consult a specialist behavioral therapist when behavior problems occur. As pointed out by Blackwell et al. [21], although a standard therapy helped reduce dogs’ separation anxiety, an individualized behavior therapy was more successful.

Besides education of the new owners, many authors [2, 44–48] and the Directive 2010/63/EU [1] emphasize the importance of pre-adoption training programs for the dogs. Applicable methods for such training are given by [2, 46–48]. Among them are the socialization of puppies with humans and conspecifics during the “sensitive phase” of 3 to 14 weeks of age; habituation to various environmental and everyday stimuli (sounds, e.g., from audio CDs; objects); learning of leash-behavior and basic commands; and preparation for housetraining through appropriate housing conditions. Such programs are beneficial not only for the dogs but also for staff and experimenters [46, 49].

## Conclusion

The majority of the dogs proved to be well adapted to their new life situation as determined in the observational test 6 weeks and in the interviews 1 and 12 weeks after rehoming. The results demonstrated good bonding between dog and new owner. For many of the parameters, the assessments from the observational test showed correlations with those from the preceding behavior tests and from the interviews. The analysis of influencing factors confirmed the results from the analyses of the behavior tests and the interviews by Döring et al. [10]. The development of separation problems could not be predicted by the preceding behavior tests or the interviews.

## Recommendations

Based on the results of this study, with consideration of data from Döring et al. [10], we make the following recommendations:

1. Former laboratory beagles adapt well to their new life situation. The rehoming can generally be seen as very positive and thus should be facilitated.
2. Collaboration with specialized rehoming organizations is recommendable.
3. New owners should be carefully selected and educated by the rehoming organization. Even first-time dog owners may be suitable candidates. The presence of a partner dog is not necessary.
4. Regular obedience training with positive reinforcement is recommendable.
5. Owners should be informed about probable behavior problems (housetraining, separation problems, fear of sounds and objects) and receive advice on how to best prevent them. They should receive contact information for competent behavioral therapists.
6. Laboratory beagles may be placed in households with children. Although laboratory beagles are very tolerant and rarely aggressive, safety rules between dog and child with regard to bite prevention must be observed.
7. The dogs can be rehomed regardless of age. Although puppies under 6 months of age received the best scores, no age differences existed in the dogs’ adaptation capability. Especially older dogs (> 2.5 years of age) also received high scores.
8. Research facilities should carefully select the breeders in order to purchase friendly and fearless, i.e., well-socialized and habituated, dogs.
9. A behavior test in the research facility has only low to moderate predictive power. It is best suited to predict the behavior towards unknown persons (visitors).

## Supporting information

S1 Table Selected test parts of the behavior tests Test 1 and Test 2 and the observational test and corresponding parameters asked in the phone interviews Interview 1 and Interview 2. The behaviors were defined (for Tests 1 and 2 and Interviews 1 and 2 see Döring et al. [10], for the observational test see Table 2) and assessed using behavior scores. These selected test parts and parameters were used for the calculation of correlations (Table 3 and S5 Table) and for the calculation of the personality score of each individual dog (mean of the behavior scores of these selected test parts/parameters). The personality scores were used to analyze factors that could influence the behavior by applying mixed regression models (S6 Table).
(DOCX)

S2 Table Phone interviews with the new owners 1 week (Interview 1) and 12 weeks (Interview 2) after adoption. Description of the questions asked, definition of the behavior categories, and results (percentage and—in brackets—number of dogs that showed the behavior), complete data.
* In the first week, only 43 owners took their dog to a pedestrian zone; hence, we omitted these data.
(DOCX)

S3 Table Observational test: Test parts in the order they were performed and description of the test parts, definition of the behavior categories, behavior scores and test results (percentage and—in brackets—number of dogs that showed the behavior). Scores were only given in test parts comparable with those of the behavior tests 1 and 2 (described in Döring et al. [10], S1 Table), complete data.
* Test parts were not conducted with dogs that panicked. Ratchet sound not conducted with 11 dogs, hide-and-seek not conducted with 5 dogs, vacuum cleaner not conducted with 10 dogs (the owners said that they used the vacuum cleaner only when the dog was absent because of his/her fear reactions).
** Every dog was offered the same type of treat, which the test person brought with her.
Signals of fear = submissive or crouched body posture, tucking tail, “stress signals” (freezing, shivering, urination, defecation), “calming signals” (muzzle licking, paw lifting, yawning).
(DOCX)

S4 Table Mean scores and SEM (in brackets) in the observational test (6 weeks after adoption, n = 74). * Test parts with asterisks were conducted by the owner. The other test parts were conducted by the test person in the presence of the owner.
Playing, chasing, feeding and obedience were not scored except for body language. Object and noise subsequent reactions were not scored for body language. Object: mean of scores of three object tests: vacuum cleaner, garbage can and balloon.
(DOCX)

S5 Table Correlations in dogs ≥2 years of age. Correlation of behavior scores between observational test (conducted 6 weeks after adoption) and behavior tests (Test 1 and Test 2, conducted before and 6 weeks after adoption, respectively) and between observational test and phone interviews (Interview 1 and Interview 2, conducted 1 and 12 weeks after adoption, respectively) and correlation of body language scores between observational test and both behavior tests.
^a)^ Behavior was not scored, but presence/absence of behavior was determined (e.g., playing: Does dog play in Test 1 and Test 2, yes or no?).
^b)^ For the observational test, we calculated the mean from three object test parts: vacuum cleaner, garbage can and balloon.
^c)^ For Tests 1 and 2, mean values were calculated from four examinations: ears, mouth, legs and auscultation.
Colors:
White: no correlation (<0.2).
Light gray: low correlation (0.2 to <0.4).
Dark gray: moderate correlation (0.4 to <0.7).
Blue: high correlation (≥0.7).
(DOCX)

S6 Table Results from the analysis of influencing factors based on the personality scores (mean of scores) in the observational test (“main model” = mixed regression model; “explorative model” = mixed regression model with Forward AIC Selection). ^a)^ According to the variables in Interview 1.
^b)^ “Punishment” consisted mainly in the loud commands “Pfui”, “Aus”, “Nein”, (which mean “Leave it”, “Drop it”, “No”, respectively) to interrupt the behavior (86% of owners in Interview 1, 95% of owners in Interview 2) or in ignoring (8% in Interview 1, 9% in Interview 2). Physical punishment was applied by only 1% (Interview 1) or 2% (Interview 2) of owners.
^c)^ Inclusion of age as smooth terms, thus with estimated degrees of freedom (edf).
^d)^ According to the variables in Interview 2.
(DOCX)

S7 Table Data of box plots (Figs 2–5) and explorative models. Breeder: All categories of commercial breeders were combined to “breeder”*.
Residential area: The categories “city” and “suburb” were combined to “urban”*.
Family members: The categories without children were combined to “no child”*; families with grandchildren were excluded.
Punishment: To build these binary categories, the categories “medium” and “frequently” (definitions in Döring et al. [10]) were combined to “frequently”, the categories “none” and “rarely” were combined to “rarely”.
Rewarding: To build these binary categories, the categories “medium” and “frequently” (definitions in Döring et al. [10]) were combined to “frequently”.
I1 = Using the variables of Interview 1; I2 = using the variables of Interview 2; because there were different numbers of owners in Interviews 1 and 2 regarding the attendance of dog classes, applying obedience training and frequency of rewarding or punishment, the explorative model was calculated twice.
* Categories with asterisk were used for generating box plots (Figs 2–5).
(DOCX)
