# Supplementary material for: How do rehomed laboratory beagles behave in everyday situations? Results from an observational test and a survey of new owners
Source: PLoS One. 2017 Jul 25;12(7):e0181303. doi: 10.1371/journal.pone.0181303 (PMC5526562; doi:10.1371/journal.pone.0181303)
Supplement: S1 Table — The behaviors were defined (for Tests 1 and 2 and Interviews 1 and 2 see Döring et al. [10], for the observational test see Table 2) and assessed using behavior scores. These selected test parts and parameters were used for the calculation of correlations (Table 3 and S5 Table) and for the calculation of the personality score of each individual dog (mean of the behavior scores of these selected test parts/parameters). The personality scores were used to analyze factors that could influence the behavior by applying mixed regression models (S6 Table). (DOCX) [file pone.0181303.s001.docx]

| **Topic** | **Behavior tests:**  **Test 1 and 2** | **Phone interviews: Interview 1 and 2** | **Observational test** |
| --- | --- | --- | --- |
| Behavior when left alone | Isolation  Dog is 90 seconds alone in the test arena. | Isolation  Dog is left alone in the home. Behavior of the dog is being observed by the owner and/or a neighbor by listening and watching through a window or glass door. | - |
| Behavior towards humans | Contact  Test person enters and stands motionless for 60 seconds. | Contact with visitor  A person unknown to the dog enters the home. | Contact with visitor  The test person rings the door bell and enters the home. |
|  | Luring  Test person squats down and claps hands, dog is petted when he/she comes to the test person. | Luring  Reaction of the dog when being lured by the owner. | Luring  The owner calls the dog. |
|  | Following  Test person passes the dog and strides once around the test arena, ignoring the dog. | - | - |
| Behavior when being importuned | Provocation  Test person grips the muzzle and holds it shut for 10 seconds with one hand, the other hand is placed on the dog’s neck. | Provocation  Behavior of the dog when the owner leans over the dog, when the owner carries the dog on the arms and when the owner pushes the dog. (Mean of scores of these three situations) | - |
| Behavior when being confronted with an object: First reaction | Unknown object  First reaction  Test person shakes an empty plastic bag open, twists the bag and places the bag on the floor. | Object  First reaction  Reaction of the dog to objects. (Question: Did you notice that your dog shows fear or unease when he/she sees certain objects?) | Object  First reaction  Object: vacuum cleaner  The owner takes out a vacuum cleaner and turns it on.  Object: garbage can  The test person pulls a garbage can past the dog.  Object: balloon  The test person moves a balloon on a string back and forth.  (Mean of the scores of these three situations) |
| Subsequent reaction | Unknown object  Subsequent reaction | Object  Subsequent reaction | Object  Subsequent reaction  (Mean of the scores of the three situations vacuum cleaner, garbage can and balloon) |
| Behavior when being confronted with a noise:  First reaction | Noise  First reaction  Test person rings a bicycle bell, her back facing the dog. | Noise  First reaction  Reaction of the dog to noises. (Question: Did you notice that your dog shows fear or unease when he/she hears certain noises?) | Noise  First reaction  The test person makes a loud, unfamiliar noise with a ratchet. |
| Subsequent reaction | Noise  Subsequent reaction | Noise  Subsequent reaction | Noise  Subsequent reaction |
| Behavior when the dog is examined | Examination  The test person squats down, examines both of the dog’s ears, opens his/her mouth, lifts all of the dog’s legs one after the other and determines the heart rate with a stethoscope. (Mean score of these four examinations) | Examination by the owner  Behavior when being fixated and examined by the owner. | Examination by the owner  The owner lifts the dog’s lip and examines the teeth, looks into both ears and strokes forelegs and hind legs with one hand. |
| Behavior when a collar /harnish is put on the dog | Placing collar and leash  Test person places a collar and a leash on the dog. | Placing collar and leash  Behavior when owner places collar/harness and leash on dog. | Placing collar and leash  The owner places collar and leash on the dog. |
| Behavior when the dog is led on a leash | Leash-behavior  Test person gets up and walks 2–4 steps with the dog then stops and takes the leash and collar off the dog. | Leash-behavior  Behavior of dog while being walked outdoors on a leash. | Leash-behavior  The owner leaves the house with the leashed dog. |
| Behavior when the dog encounters another dog | - | Other dogs  Behavior of the dog when meeting other dogs while on a walk or when other dogs visit the dog owner’s home. | Unknown female dog  The test dog “Lauri” (female Beauceron, 6 years old) is led past the dog at about 2 m. distance. |
