# Supplementary material for: How do rehomed laboratory beagles behave in everyday situations? Results from an observational test and a survey of new owners
Source: PLoS One. 2017 Jul 25;12(7):e0181303. doi: 10.1371/journal.pone.0181303 (PMC5526562; doi:10.1371/journal.pone.0181303)
Supplement: S2 Table — Description of the questions asked, definition of the behavior categories, and results (percentage and—in brackets—number of dogs that showed the behavior), complete data. * In the first week, only 43 owners took their dog to a pedestrian zone; hence, we omitted these data. (DOCX) [file pone.0181303.s002.docx]

| **Parameters and behavior categories** | **Description/Definition** | **Interview 1** | **Interview 2** |
| --- | --- | --- | --- |
| **Behavior towards the new owner** | |  |  |
| **Owner petting the dog** | *Behavior of the dog when being petted by the owner* | (n = 142) | (n = 124) |
| enjoys | dog wags tail/ rolls on back/ closes eyes/ rubs him-/herself against owner and so forth | 89% (127) | 94% (116) |
| acceptance | dog tolerates the situation with tensed body | 11% (15) | 6% (8) |
| slight withdrawal | dog tries to withdraw (movement of head or body) | 0% (0) | 0% (0) |
| moves away | dog moves away from owner, owner cannot pet the dog | 0% (0) | 0% (0) |
| aggression | dog barks or growls or bares teeth or snaps | 0% (0) | 0% (0) |
| **Dog seeks contact with owner** | *Dog seeks contact with owner by touching the owner with the snout/ jumping up/ fixating on the owner/ jumping on the owner’s lap/ scratching the owner / whining/ barking/ snuggling up to the owner* | (n = 136) | (n = 123) |
| frequently | dog seeks contact more than 8 times a day | 39% (53) | 52% (64) |
| sometimes | dog seeks contact 4–8 times a day | 32% (44) | 33% (40) |
| rarely | dog seeks contact 1–3 times a day | 12% (16) | 10% (12) |
| not at all | dog does not seek contact with the owner | 17% (23) | 6% (7) |
| **Whereabouts of the dog during the day** | *Whereabouts of the dog during the day with regard to the person to whom the dog is most attached* | (n = 136) | (n = 124) |
| follows persistently | dog persistently follows the owner, stays within 2 m of the owner all day | 24% (32) | 25% (31) |
| stays nearby (balanced) | dog usually stays in the same room as the owner, checks where the owner is, does spend some time in other rooms or in the garden | 54% (74) | 57% (71) |
| fear and avoidance | dog tries to stay away from the owner | 6% (8) | 5% (6) |
| does something else | dog does not follow the owner around | 16% (22) | 13% (16) |
| **Whereabouts of the dog at night** | *Whereabouts of the dog at night with regard to the person to whom the dog is most attached* | (n = 136) | (n = 126) |
| bedroom | dog chooses to sleep in the same room as the owner | 34% (46) | 35% (44) |
| different room | dog chooses to sleep in a different room than the owner | 23% (31) | 24% (30) |
| has no choice | dog cannot choose sleeping area | 43% (59) | 41% (52) |
| **Grooming tasks performed by owner** | *Behavior of the dog when being brushed or washed by the owner* | (n = 128) | (n = 123) |
| acceptance | dog tolerates the situation | 90% (115) | 93% (114) |
| slight withdrawal | dog tries to withdraw (movement of head or body) | 1% (1) | 4% (5) |
| freeing | dog frees him-/herself from the owner’s fixation | 3% (4) | 2% (2) |
| moves away | dog moves away from owner, owner cannot perform tasks | 6% (8) | 2% (2) |
| aggression | dog barks or growls or bares teeth or snaps | 0% (0) | 0% (0) |
| **Behavior towards family members** | | | |
| **Greeting other family members** | *Behavior of the dog when a family member other than the owner enters the residence* | (n = 127) | (n = 121) |
| friendly contact | dog walks toward the family member in a speedy manner with a relaxed body posture and licks/ sniffs/ jumps up | 72% (92) | 86% (104) |
| cautious contact | dog hesitantly approaches the family member with signals of fear, watches person/ sniffs/ licks | 12% (15) | 7% (8) |
| fear and avoidance | dog does not approach the family member, dog moves away when the family member approaches and shows signals of fear | 4% (5) | 2% (2) |
| does something else | dog does not seek contact and shows no change of current behavior | 12% (15) | 4% (5) |
| active aggression | dog approaches the family member and barks or growls or bares teeth or snaps | 0% (0) | 1% (1) |
| defensive aggression | dog barks or growls or bares teeth or snaps when being approached by the family member | 0% (0) | 1% (1) |
| **Behavior towards family members** | *Behavior of the dog when in close proximity to family members who are over 15 years of age*  *(In case of multiple answers because of multiple family members, the worst category was applied)* | (n = 139) | (n = 123) |
| friendly contact | dog approaches the family member/ wags tail/ jumps up/ has a relaxed body posture/ responds happily to petting and/or play | 81% (112) | 89% (109) |
| cautious contact | dog approaches the family member with signals of fear | 10% (14) | 2% (2) |
| fear and avoidance | dog does not approach the family member, dog moves away when the family member approaches and shows signals of fear | 7% (10) | 7% (8) |
| does something else | dog does not seek contact and shows no change of current behavior | 1% (2) | 1% (1) |
| active aggression | dog approaches the family member and barks or growls or bares teeth or snaps | 0% (0) | 1% (1) |
| defensive aggression | dog barks or growls or bares teeth or snaps when being approached by the family member | 1% (1) | 2% (2) |
| **Behavior towards children in the family** | *Behavior of the dog towards children (up to 15 years of age) living in the same household when they approach the dog to pet him/ her* | (n = 69) | (n = 62) |
| friendly contact | dog approaches the child/ wags tail/ jumps up/ has a relaxed body posture/ wants to be petted and/or wants to engage in play | 68% (47) | 77% (48) |
| cautious contact | dog approaches the child with signals of fear | 13% (9) | 11% (7) |
| fear and avoidance | dog does not approach the child, dog moves away when the child approaches and shows signals of fear | 12% (8) | 5% (3) |
| does something else | dog does not seek contact and shows no change of current behavior | 3% (2) | 2% (1) |
| active aggression | dog approaches the child and barks or growls or bares teeth or snaps | 0% (0) | 0% (0) |
| defensive aggression | dog barks or growls or bares teeth or snaps when being approached by the child | 4% (3) | 3% (2) |
| chasing behavior | dog chases the child | 0% (0) | 2% (1) |
| **Behavior towards partner dogs** | *Behavior of dog towards the other family dog(s)* | (n = 61) | (n = 45) |
| friendly contact | dog wags tail, has relaxed body posture, plays with the other dog | 72% (44) | 84% (38) |
| cautious contact | dog hesitantly approaches the other dog with signals of fear | 2% (1) | 4% (2) |
| fear and avoidance | dog does not approach the other dog, dog moves away when the other dog approaches him/her and shows signals of fear | 3% (2) | 0% (0) |
| does something else | dog does not seek contact and shows no change of current behavior | 5% (3) | 2% (1) |
| assertive demonstration | head held high, legs extended, tail pointing up, stiff and tense body posture | 11% (7) | 9% (4) |
| active aggression | dog approaches the other dog and barks or growls or bares teeth or snaps | 0% (0) | 0% (0) |
| defensive aggression | dog barks or growls or bares teeth or snaps when being approached by the other dog | 0% (0) | 0% (0) |
| resource-oriented behavior | dog protects his/her own food/ toy/ resting area and/or challenges the other dog for his/her food/ toy/ resting area with or without threat or aggressive behavior | 7% (4) | 0% (0) |
| **Behavior towards owner’s cat** | *Behavior of dog towards the family cat(s)* | (n = 38) | (n = 29) |
| friendly contact | dog sniffs or looks at cat, wags tail, has relaxed body posture, respects defensive behavior of the cat by withdrawing | 58% (22) | 59% (17) |
| cautious contact | dog hesitantly approaches the cat with signals of fear | 5% (2) | 0% (0) |
| fear and avoidance | dog does not approach the cat, dog moves away when the cat approaches him/her and shows signals of fear | 5% (2) | 0% (0) |
| does something else | dog does not seek contact and shows no change of current behavior | 26% (10) | 17% (5) |
| active aggression | dog approaches the cat and barks or growls or bares teeth or snaps | 3% (1) | 10% (3) |
| defensive aggression | dog barks or growls or bares teeth or snaps when being approached by the cat | 0% (0) | 0% (0) |
| chasing behavior | dog chases the cat | 3% (1) | 14% (4) |
| **Behavior towards strangers and in various situations** | | | |
| **Behavior towards unknown children** | *Behavior of the dog towards children (up to 15 years of age) unknown to the dog when they approach the dog to pet him/her* | (n = 36) | (n = 48) |
| friendly contact | dog approaches the child/ wags tail/ jumps up/ has a relaxed body posture/ wants to be petted and/or wants to engage in play | 56% (20) | 44% (21) |
| cautious contact | dog approaches the child with signals of fear | 22% (8) | 10% (5) |
| fear and avoidance | dog does not approach the child, dog moves away when the child approaches him/her and shows signals of fear | 11% (4) | 19% (9) |
| does something else | dog does not seek contact and shows no change of current behavior | 8% (3) | 15% (7) |
| active aggression | dog approaches the child and barks or growls or bares teeth or snaps | 3% (1) | 0% (0) |
| defensive aggression | dog barks or growls or bares teeth or snaps when being approached by the child | 0% (0) | 13% (6) |
| chasing behavior | dog chases the child | 0% (0) | 0% (0) |
| **Contact with passerby** | *Encounter with a neutral person (passerby) unknown to the dog while on a walk* | (n = 138) | (n = 121) |
| friendly contact | dog walks toward the person in a speedy manner with a relaxed body posture and licks/ sniffs/ jumps up | 41% (56) | 50% (60) |
| cautious contact | dog hesitantly approaches the person with signals of fear, watches person/ sniffs/ licks | 19% (26) | 7% (9) |
| fear and avoidance | dog does not approach the person, dog moves away when the person approaches him/her and shows signals of fear | 22% (31) | 17% (20) |
| does something else | dog does not seek contact and shows no change of current behavior | 15% (21) | 23% (28) |
| active aggression | dog approaches the person and barks or growls or bares teeth or snaps | 1% (1) | 1% (1) |
| defensive aggression | dog barks or growls or bares teeth or snaps when being approached by the person | 2% (3) | 2% (3) |
| **Examination by a veterinarian** | *Behavior of the dog when being fixated, cared for, brushed or examined by a veterinarian* | (n = 43) | (n = 83) |
| acceptance | dog tolerates the situation | 93% (40) | 89% (74) |
| slight withdrawal | dog tries to withdraw (movement of head or body) | 2% (1) | 5% (4) |
| freeing | dog frees him-/herself from the veterinarian’s fixation | 0% (0) | 0% (0) |
| moves away | dog moves away from veterinarian, veterinarian cannot perform examination | 2% (1) | 4% (3) |
| aggression | dog barks or growls or bares teeth or snaps at the veterinarian | 2% (1) | 2% (2) |
| **Car ride** | *Behavior of the dog during a car ride* | (n = 124) | (n = 118) |
| relaxed | dog is calm with relaxed body posture and without signals of fear | 75% (93) | 75% (88) |
| agitated | dog shows agitation by increased movements/ panting/ pulling on leash | 7% (9) | 13% (15) |
| fearful | dog shows one of the following behaviors: submissive or crouched body posture, tucking tail, calming signal, shivering, strong unrest | 8% (10) | 6% (7) |
| panicking | dog shows at least two of the following behaviors: submissive or crouched body posture, tucking tail, shivering, whining or yelping, trying to escape, struggling, strong unrest, uncontrolled movements, elimination, freezing | 10% (12) | 7% (8) |
| sickness | dog salivates or heaves or vomits | 22% (27) | 26% (31) |
| **Unfamiliar walk** | *Behavior of the dog during a walk in an unfamiliar area* | (n = 123) | (n = 119) |
| relaxed | dog is calm with relaxed body posture and without signals of fear | 46% (57) | 51% (61) |
| agitated | dog shows agitation by increased movements/ panting/ pulling on leash | 30% (37) | 40% (48) |
| fearful | dog shows one of the following behaviors: submissive or crouched body posture, tucking tail, calming signal, shivering, strong unrest | 14% (17) | 8% (9) |
| panicking | dog shows at least two of the following behaviors: submissive or crouched body posture, tucking tail, shivering, whining or yelping, trying to escape, struggling, strong unrest, uncontrolled movements, elimination, freezing | 10% (12) | 1% (1) |
| **Pedestrian zone** | *Behavior of the dog in a pedestrian zone* | ***** | (n = 83) |
| relaxed | dog is calm with relaxed body posture and without signals of fear |  | 39% (32) |
| agitated | dog shows agitation by increased movements/ panting/ pulling on leash |  | 29% (24) |
| fearful | dog shows one of the following behaviors: submissive or crouched body posture, tucking tail, calming signal, shivering, strong unrest |  | 16% (13) |
| panicking | dog shows at least two of the following behaviors: submissive or crouched body posture, tucking tail, shivering, whining or yelping, trying to escape, struggling, strong unrest, uncontrolled movements, elimination, freezing |  | 17% (14) |
