# Supplementary material for: How do rehomed laboratory beagles behave in everyday situations? Results from an observational test and a survey of new owners
Source: PLoS One. 2017 Jul 25;12(7):e0181303. doi: 10.1371/journal.pone.0181303 (PMC5526562; doi:10.1371/journal.pone.0181303)
Supplement: S3 Table — Scores were only given in test parts comparable with those of the behavior tests 1 and 2 (described in Döring et al. [10], S1 Table), complete data. * Test parts were not conducted with dogs that panicked. Ratchet sound not conducted with 11 dogs, hide-and-seek not conducted with 5 dogs, vacuum cleaner not conducted with 10 dogs (the owners said that they used the vacuum cleaner only when the dog was absent because of his/her fear reactions). ** Every dog was offered the same type of treat, which the test person brought with her. Signals of fear = submissive or crouched body posture, tucking tail, “stress signals” (freezing, shivering, urination, defecation), “calming signals” (muzzle licking, paw lifting, yawning). (DOCX) [file pone.0181303.s003.docx]

| **Test parts and behavior categories** | **Description/ Definition** | **Score** | **Results** |
| --- | --- | --- | --- |
| **Contact with visitor** | *The test person goes outside and rings the door bell, the owner opens the door, and the test person enters the residence (without being greeted by the owner) while the reaction of the dog is filmed from a tripod.* |  | (n = 56) |
| friendly contact | dog walks toward the person in a speedy manner with a relaxed body posture and licks/ sniffs/ jumps up | 3 | 36% (20) |
| cautious contact | dog hesitantly approaches the person with signals of fear, watches person/ sniffs/ licks | 2 | 23% (13) |
| fear and avoidance | dog does not approach the person; dog moves away when the person approaches him/her and shows signals of fear | 0 | 27% (15) |
| does something else | dog does not seek contact and shows no change of current behavior | 2 | 14% (8) |
| active aggression | dog approaches the person and barks or growls or bares teeth or snaps | 0 | 0% (0) |
| defensive aggression | dog barks or growls or bares teeth or snaps when being approached by the person | 0 | 0% (0) |
| **Luring** | *The owner calls the dog in his/her own way from a distance of at least 3 m.* |  | (n = 73) |
| comes immediately | dog comes directly to the owner without hesitation | 3 | 60% (44) |
| comes hesitantly | dog approaches the owner slowly and cautiously | 2 | 19% (14) |
| does not come | dog does not approach the owner | 0 | 21% (15) |
| **Examination by the owner** | *The owner lifts the dog’s lip and examines the teeth, looks into both ears and strokes forelegs and hind legs with one hand.* |  | (n = 71) |
| acceptance | dog tolerates the situation | 3 | 92% (65) |
| slight withdrawal | dog tries to withdraw (movement of head or body) | 2 | 6% (4) |
| moves away | dog moves away from the owner, owner cannot perform examination | 0 | 3% (2) |
| aggression | dog barks or growls or bares teeth or snaps | 0 | 0% (0) |
| **Playing** | *The owner offers the dog a toy of his/her choice and encourages the dog to play.* |  | (n = 69) |
| plays | dog follows the toy and/or picks up the toy with his/her mouth | - | 58% (40) |
| does not play | dog does not engage in the play and does something else | - | 42% (29) |
| **Noise**  **First reaction** | *The test person makes a loud, unfamiliar noise with a ratchet.* |  | (n = 57)* |
| is relaxed | dog does not flinch and does not move back | 3 | 51% (29) |
| gets startled | dog flinches | 1 | 35% (20) |
| gets frightened | dog cringes or moves back | 0 | 14% (8) |
| **Subsequent reaction** | *The test person puts the ratchet on the floor for approx. 15 seconds.* |  |  |
| makes contact | dog approaches and watches object and/or makes contact with his/her snout | 3 | 74% (42) |
| is relaxed | dog shows no reaction | 3 | 11% (6) |
| fear and avoidance | dog moves back and/or stays at a distance, shows signals of fear | 0 | 16% (9) |
| **First hide-and-seek** | *The owner hides without the dog noticing and calls the dog.* |  | (n = 62)* |
| seeks immediately | dog seeks and finds the owner without hesitation and straightaway | - | 63% (39) |
| seeks hesitantly | dog seeks and finds the owner with slow and hesitant search behavior | - | 23% (14) |
| does dot seek | dog does something else | - | 15% (9) |
| **Second hide-and-seek** | *The test part is repeated after the dog found the owner.* |  | (n = 54)* |
| seeks immediately | dog seeks and finds the owner without hesitation and straightaway | - | 63% (34) |
| seeks hesitantly | dog seeks and finds the owner with slow and hesitant search behavior | - | 19% (10) |
| does dot seek | dog does something else | - | 19% (10) |
| **Faster finding** |  |  | (n = 46) |
| yes | dog finds the owner faster during repetition | - | 70% (32) |
| no | dog needs the same time or longer to find the owner during repetition | - | 30% (14) |
| **Object (vacuum cleaner)**  **First reaction** | *The owner takes out a vacuum cleaner and turns it on.* |  | (n = 56)* |
| is relaxed | dog does not flinch or move back | 3 | 39% (22) |
| gets startled | dog flinches | 1 | 45% (25) |
| gets frightened | dog cringes or moves back | 0 | 16% (9) |
| **Subsequent reaction** |  |  |  |
| makes contact | dog approaches and watches object and/or makes contact with his/her snout | 3 | 27% (15) |
| is relaxed | dog shows no reaction | 3 | 27% (15) |
| fear and avoidance | dog moves back and/or stays at a distance, shows signals of fear | 0 | 46% (26) |
| **Behavior towards partner dog** | *Behavior of the dog towards the other dog (during the entire test)* |  | (n = 25) |
| friendly contact | dog is wagging his/her tail, has relaxed body posture, plays with the other dog | - | 76% (19) |
| cautious contact | dog hesitantly approaches the other dog with signals of fear | - | 0% (0) |
| fear and avoidance | dog does not approach the other dog, dog moves away when being approached by the other dog and shows signals of fear | - | 0% (0) |
| does something else | dog does not seek contact and shows no change of current behavior | - | 24% (6) |
| assertive demonstration | head held high, legs extended, tail pointing up, stiff and tense body posture | - | 0% (0) |
| active aggression | dog approaches the other dog and barks or growls or bares teeth or snaps | - | 0% (0) |
| defensive aggression | dog barks or growls or bares teeth or snaps when being approached by the other dog | - | 0% (0) |
| resource-oriented behavior | dog protects his/her food/ toy/ resting area and/or challenges the other dog for his/her food/ toy/ resting area with or without threat or aggressive behavior | - | 0% (0) |
| **Placing collar and leash** | *The owner places collar and leash on the dog.* |  | (n = 67) |
| acceptance | dog tolerates the situation | 3 | 85% (57) |
| slight withdrawal | dog tries to withdraw (movement of head or body) | 2 | 4% (3) |
| moves away | dog moves away from owner, owner cannot place collar | 0 | 10% (7) |
| aggression | dog barks or growls or bares teeth or snaps | 0 | 0% (0) |
| **Leash-behavior** | *The owner leaves the house with the leashed dog* |  | (n = 71) |
| follows along | dog follows along without the dog or the owner pulling on the leash | 3 | 80% (57) |
| moves after pulling | dog follows along only when the owner pulls on the leash | 1 | 3% (2) |
| pulls on the leash | dog pulls on the leash | 1 | 11% (8) |
| does not walk on the leash | even after pulling on the leash, the owner cannot make the dog walk, dog stops, sits or lies down | 0 | 6% (4) |
| **Object (garbage can)**  **First reaction** | *The test person pulls a garbage can past the dog at a distance of max. 2 m.* |  | (n = 57) |
| is relaxed | dog does not flinch or move back | 3 | 54% (31) |
| gets startled | dog flinches | 1 | 35% (20) |
| gets frightened | dog cringes or moves back | 0 | 11% (6) |
| **Subsequent reaction** |  |  |  |
| makes contact | dog approaches and watches object and/or makes contact with his/her snout | 3 | 37% (21) |
| is relaxed | dog shows no reaction | 3 | 35% (20) |
| fear and avoidance | dog moves back and/or stays at a distance, shows signals of fear | 0 | 28% (16) |
| **Standing or walking at a busy street** | *The owner takes the dog to a busy street, stops and walks along the street for at least 2 minutes until a bus, a truck or another vehicle with a noisy engine passes.* |  | (n = 68) |
| **Walking at the busy street (no vehicle)** | *The dog is observed while walking along the street when no cars or trucks are passing (but bicycles, pedestrians, buggies, etc. may pass).* |  |  |
| **First reaction** |  |  |  |
| is relaxed | dog does not flinch or move back | - | 82% (56) |
| gets startled | dog flinches | - | 12% (8) |
| gets frightened | dog cringes or moves back | - | 6% (4) |
| **Subsequent reaction** |  |  |  |
| makes contact | dog approaches the street or the passing object and/or watches object | - | 56% (38) |
| is relaxed | dog shows no reaction | - | 31% (21) |
| fear and avoidance | dog moves back and/or stays at a distance, shows signals of fear | - | 13% (9) |
| **Car** | *A car passes.* |  | (n = 62) |
| **First reaction** |  |  |  |
| is relaxed | dog does not flinch or move back | - | 92% (57) |
| gets startled | dog flinches | - | 8% (5) |
| gets frightened | dog cringes or moves back | - | 0% (0) |
| **Subsequent reaction** |  |  |  |
| makes contact | dog approaches the street and/or follows the car with the eyes | - | 10% (6) |
| is relaxed | dog shows no reaction | - | 89% (55) |
| fear and avoidance | dog moves back and/or stays at a distance, shows signals of fear | - | 2% (1) |
| **Truck** | *A truck passes.* |  | (n = 34) |
| **First reaction** |  |  |  |
| is relaxed | dog does not flinch or move back | - | 85% (29) |
| gets startled | dog flinches | - | 6% (2) |
| gets frightened | dog cringes or moves back | - | 9% (3) |
| **Subsequent reaction** |  |  |  |
| makes contact | dog approaches the street and/or follows the truck with the eyes | - | 9% (3) |
| is relaxed | dog shows no reaction | - | 76% (26) |
| fear and avoidance | dog moves back and/or stays at a distance, shows signals of fear | - | 15% (5) |
| **Passerby** | *Random encounter with a neutral person (passerby) unknown to the dog (no standardized test person)* |  | (n = 45) |
| friendly contact | dog walks toward the person in a speedy manner with a relaxed body posture and licks/ sniffs/ jumps up | - | 36% (16) |
| cautious contact | dog hesitantly approaches the person with signals of fear, watches person/ sniffs/ licks | - | 7% (3) |
| fear and avoidance | dog does not approach the person, dog moves away when being approached by the person and shows signals of fear | - | 16% (7) |
| does something else | dog does not seek contact and shows no change of current behavior | - | 40% (18) |
| active aggression | dog approaches the person and barks or growls or bares teeth or snaps | - | 2% (1) |
| defensive aggression | dog barks or growls or bares teeth or snaps when being approached by the person | - | 0% (0) |
| **Staircase** | *The owner takes the dog up and down a staircase of at least 5 stairs.* |  | (n = 52) |
| goes immediately | dog walks up and down the stairs without hesitation | - | 90% (47) |
| goes hesitantly | dog walks slowly and hesitantly up and down the stairs | - | 6% (3) |
| does not go | dog does not step on the staircase | - | 4% (2) |
| **Object (balloon)**  **First reaction** | *The test person moves a balloon on a string back and forth in front of the dog at a distance of approx. 0.5 m.* |  | (n = 68) |
| is relaxed | dog does not flinch or move back | 3 | 57% (39) |
| gets startled | dog flinches | 1 | 32% (22) |
| gets frightened | dog cringes or moves back | 0 | 10% (7) |
| **Subsequent reaction** |  |  |  |
| makes contact | dog approaches and/or watches object and/or tries to make contact with his/her snout (touching the balloon was not allowed) | 3 | 65% (44) |
| is relaxed | dog shows no reaction | 3 | 7% (5) |
| fear and avoidance | dog moves back and/or stays at a distance, shows signals of fear | 0 | 28% (19) |
| **Chasing** | *The test person pulls a toy ball made of fur rapidly past the dog at a distance of approx. 0.5 m.* |  | (n = 55) |
| follows | dog chases after the object | - | 60% (33) |
| does not follow | dog does not follow the object and does something else | - | 40% (22) |
| **Unknown female dog** | *The test dog “Lauri” (female Beauceron, 6 years old) is led past the dog at about 2 m distance.* |  | (n = 64) |
| friendly contact | dog is wagging his/her tail, has relaxed body posture, plays with the other dog | 3 | 66% (42) |
| cautious contact | dog hesitantly approaches the other dog with signals of fear | 1 | 20% (13) |
| fear and avoidance | dog does not approach the other dog, dog moves away when being approached by the other dog and shows signals of fear | 0 | 8% (5) |
| does something else | dog does not seek contact and shows no change of current behavior | 2 | 0% (0) |
| assertive demonstration | head held high, legs extended, tail pointing up, stiff and tense body posture | 1 | 3% (2) |
| active aggression | dog approaches the other dog and barks or growls or bares teeth or snaps | 0 | 2% (1) |
| defensive aggression | dog barks or growls or bares teeth or snaps when being approached by the other dog | 0 | 2% (1) |
| **Obedience** | *The owner calls the dog and commands “sit”* |  | (n = 54) |
| obeys immediately | dog obeys the command without hesitation | - | 65% (35) |
| obeys hesitantly | dog obeys the command with hesitation and/or after several prompts | - | 9% (5) |
| does not obey | dog does not obey the command | - | 26% (14) |
| **Feeding out of hand** | *The test person offers the dog a treat out of her hand.*** |  | (n = 48) |
| eats out of hand | dog eats the food being offered out of the hand | - | 85% (41) |
| does not eat out of hand | dog does not eat the food offered out of the hand | - | 15% (7) |
