# Supplementary material for: How do rehomed laboratory beagles behave in everyday situations? Results from an observational test and a survey of new owners
Source: PLoS One. 2017 Jul 25;12(7):e0181303. doi: 10.1371/journal.pone.0181303 (PMC5526562; doi:10.1371/journal.pone.0181303)
Supplement: S4 Table — * Test parts with asterisks were conducted by the owner. The other test parts were conducted by the test person in the presence of the owner. Playing, chasing, feeding and obedience were not scored except for body language. Object and noise subsequent reactions were not scored for body language. Object: mean of scores of three object tests: vacuum cleaner, garbage can and balloon. (DOCX) [file pone.0181303.s004.docx]

| **Parameter** | **Behavior score** | **Body language score** |
| --- | --- | --- |
| Contact (visitor) | 1.82 (0.16) | 2.50 (0.09) |
| Luring* | 2.19 (0.14) | 2.69 (0.06) |
| Playing* | - | 2.83 (0.05) |
| Chasing | - | 2.75 (0.06) |
| Object 1st reaction | 1.86 (0.11) | 2.52 (0.06) |
| Object 2nd reaction | 1.96 (0.13) | - |
| Noise 1st reaction | 1.88 (0.16) | 2.49 (0.08) |
| Noise 2nd reaction | 2.53 (0.15) | - |
| Examination* | 2.82 (0.08) | 2.55 (0.08) |
| Collar* | 2.64 (0.11) | 2.49 (0.08) |
| Leash-behavior* | 2.58 (0.11) | 2.73 (0.07) |
| Feeding | - | 2.73 (0.08) |
| Unknown dog | 2.41 (0.12) | 2.66 (0.07) |
| Obedience* | - | 2.92 (0.04) |
