# Supplementary material for: How do rehomed laboratory beagles behave in everyday situations? Results from an observational test and a survey of new owners
Source: PLoS One. 2017 Jul 25;12(7):e0181303. doi: 10.1371/journal.pone.0181303 (PMC5526562; doi:10.1371/journal.pone.0181303)
Supplement: S5 Table — Correlation of behavior scores between observational test (conducted 6 weeks after adoption) and behavior tests (Test 1 and Test 2, conducted before and 6 weeks after adoption, respectively) and between observational test and phone interviews (Interview 1 and Interview 2, conducted 1 and 12 weeks after adoption, respectively) and correlation of body language scores between observational test and both behavior tests. a) Behavior was not scored, but presence/absence of behavior was determined (e.g., playing: Does dog play in Test 1 and Test 2, yes or no?). b) For the observational test, we calculated the mean from three object test parts: vacuum cleaner, garbage can and balloon. c) For Tests 1 and 2, mean values were calculated from four examinations: ears, mouth, legs and auscultation. Colors: White: no correlation (<0.2). Light gray: low correlation (0.2 to <0.4). Dark gray: moderate correlation (0.4 to <0.7). Blue: high correlation (≥0.7). (DOCX) [file pone.0181303.s005.docx]

| **Correlation with** |  | **Test 1** | **Test 2** | **Interview 1** | **Interview 2** | **Test 1** | **Test 2** |
| --- | --- | --- | --- | --- | --- | --- | --- |
| **Test situation** |  | **Behavior score** | | | | **Body language score** | |
| **Contact** | n | 27 | 36 | 31 | 32 | 27 | 36 |
| *Kruskall’s Gamma* | γ | 0.7841 | 0.7651 | 0.8407 | 0.735 | 0.4691 | 0.7087 |
| **Luring** | n | 39 | 49 | 44 | 43 | 39 | 49 |
| *Kruskall’s Gamma* | γ | 0.0722 | 0.6813 | 0.6195 | 0.6731 | 0.1888 | 0.4473 |
| **Playing** ^a)^ | n | 38 | 41 | 42 | 44 | 38 | 41 |
| *Spearman’s rank correlations* | r | 0.0679 | 0.3293 | 0.0142 | 0.3032 | 0.0825 | −0.0478 |
| **Chasing** ^a)^ | n | 29 | 34 | 32 | 32 | 29 | 34 |
| *Spearman’s rank correlations* | r | 0.0874 | 0.0913 | 0.0362 | −0.2431 | 0.0800 | 0.2898 |
| **Object, 1st reaction** ^b)^ | n | 38 | 47 | 42 | 42 | 38 | 43 |
| *Spearman’s rank correlations* | r | 0.4343 | 0.3399 | 0.0867 | 0.2865 | 0.2463 | 0.2165 |
| **Object, 2nd reaction** ^b)^ | n | 38 | 47 | 42 | 40 | - | - |
| *Kruskall’s Gamma* | γ | 0.6374 | 0.5281 | 0.2536 | 0.8338 | - | - |
| **Noise, 1st reaction** | n | 28 | 38 | 33 | 36 | 28 | 38 |
| *Spearman’s rank correlations* | r | 0.1857 | 0.2411 | 0.0865 | 0.0574 | 0.0594 | 0.3929 |
| **Noise, 2nd reaction** | n | 28 | 38 | 32 | 36 | - | - |
| *Spearman’s rank correlations* | r | 0.6655 | 0.2995 | 0.1601 | −0.2000 | - | - |
| **Examination** ^c)^ | n | 39 | 48 | 43 | 43 | 38 | 47 |
| *Spearman’s rank correlations* | r | 0.1487 | −0.0323 | −0.0494 | −0.0423 | −0.0404 | −0.0040 |
| **Placing collar** | n | 37 | 46 | 41 | 41 | 37 | 46 |
| *Spearman’s rank correlations* | r | −0.143 | −0.1903 | 0.0924 | 0.3094 | −0.2381 | 0.5059 |
| **Leash-behavior** | n | 36 | 47 | 42 | 42 | 36 | 47 |
| *Kruskall’s Gamma* | γ | -0.6667 | 0.6338 | 0.1676 | 0.6923 | 0.0521 | 0.6067 |
| **Feeding** ^a)^ | n | 24 | 24 | 26 | 28 | 24 | 24 |
| *Spearman’s rank correlations* | r | 0.2618 | 0.8452 | 0.6034 | −0.0897 | 0.3126 | 0.5291 |
| **Other dogs** | n | - | - | 39 | 40 | - | - |
| *Kruskall’s Gamma* | γ | - | - | 0.2785 | 0.5172 | - | - |
