# Supplementary material for: How do rehomed laboratory beagles behave in everyday situations? Results from an observational test and a survey of new owners
Source: PLoS One. 2017 Jul 25;12(7):e0181303. doi: 10.1371/journal.pone.0181303 (PMC5526562; doi:10.1371/journal.pone.0181303)
Supplement: S6 Table — a) According to the variables in Interview 1. b) “Punishment” consisted mainly in the loud commands “Pfui”, “Aus”, “Nein”, (which mean “Leave it”, “Drop it”, “No”, respectively) to interrupt the behavior (86% of owners in Interview 1, 95% of owners in Interview 2) or in ignoring (8% in Interview 1, 9% in Interview 2). Physical punishment was applied by only 1% (Interview 1) or 2% (Interview 2) of owners. c) Inclusion of age as smooth terms, thus with estimated degrees of freedom (edf). d) According to the variables in Interview 2. (DOCX) [file pone.0181303.s006.docx]

| **Variable** | **Estimate** | ***P*-value** |
| --- | --- | --- |
| **Main model** |  |  |
| Intercept | 2.7898 |  |
| Sex: male | −0.1495 | 0.2856 |
| Breeder: commercial | −0.7757 | 0.0113* |
| Age | 0.0755 | 0.2005 |
| **Explorative model ^a)^** |  |  |
| Intercept | 1.6281 |  |
| Garden | 0.3090 |  |
| Punishment: frequent ^b)^ | 0.2671 |  |
| >2 adults, no child | −0.2210 |  |
| At least one child | 0.2727 |  |
| Sex: male | −0.2857 |  |
| Experienced with dogs | 0.2827 |  |
| Age | edf 1.8732 ^c)^ |  |
| **Explorative model ^d)^** |  |  |
| Intercept | 1.3669 |  |
| Garden | 0.0640 |  |
| Punishment: frequent ^b)^ | 0.4670 |  |
| >2 adults, no child | 0.0030 |  |
| At least one child | −0.1014 |  |
| Dog classes | −0.4368 |  |
| Obedience training | 0.4322 |  |
| Partner dog | 0.4383 |  |
| Rewarding: frequent | 0.3224 |  |
