# Supplementary material for: How do rehomed laboratory beagles behave in everyday situations? Results from an observational test and a survey of new owners
Source: PLoS One. 2017 Jul 25;12(7):e0181303. doi: 10.1371/journal.pone.0181303 (PMC5526562; doi:10.1371/journal.pone.0181303)
Supplement: S7 Table — Breeder: All categories of commercial breeders were combined to “breeder”*. Residential area: The categories “city” and “suburb” were combined to “urban”*. Family members: The categories without children were combined to “no child”*; families with grandchildren were excluded. Punishment: To build these binary categories, the categories “medium” and “frequently” (definitions in Döring et al. [10]) were combined to “frequently”, the categories “none” and “rarely” were combined to “rarely”. Rewarding: To build these binary categories, the categories “medium” and “frequently” (definitions in Döring et al. [10]) were combined to “frequently”. I1 = Using the variables of Interview 1; I2 = using the variables of Interview 2; because there were different numbers of owners in Interviews 1 and 2 regarding the attendance of dog classes, applying obedience training and frequency of rewarding or punishment, the explorative model was calculated twice. * Categories with asterisk were used for generating box plots (Figs 2–5). (DOCX) [file pone.0181303.s007.docx]

| **Variable** | n | Mean | SEM | Median | Min | Max |
| --- | --- | --- | --- | --- | --- | --- |
| **age** |  |  |  |  |  |  |
| <0.5 years* | 7 | 2.75 | 0.07 | 2.75 | 2.44 | 3.00 |
| 0.5-2 years* | 17 | 2.26 | 0.16 | 2.60 | 1.06 | 3.00 |
| >2 years* | 50 | 2.16 | 0.09 | 2.30 | 0.60 | 3.00 |
| **sex** |  |  |  |  |  |  |
| Male* | 36 | 2.18 | 0.11 | 2.30 | 0.60 | 3.00 |
| Female* | 38 | 2.30 | 0.10 | 2.50 | 0.69 | 3.00 |
| **breeder** |  |  |  |  |  |  |
| Breeder Italy | 4 | 2.13 | 0.53 | 2.46 | 0.60 | 3.00 |
| Breeder USA | 22 | 2.40 | 0.10 | 2.54 | 1.20 | 2.93 |
| Breeder Germany 1 | 36 | 2.07 | 0.11 | 2.24 | 0.69 | 2.90 |
| Breeder Germany 2 | 3 | 1.81 | 0.22 | 1.93 | 1.38 | 2.12 |
| Breeder* | 65 | 2.17 | 0.08 | 2.30 | 0.60 | 3.00 |
| Inhouse* | 9 | 2.72 | 0.08 | 2.75 | 2.25 | 3.00 |
| **Stay in shelter** |  |  |  |  |  |  |
| none | 17 | 2.15 | 0.16 | 2.30 | 0.69 | 2.90 |
| < 1 week | 7 | 2.18 | 0.16 | 2.25 | 1.50 | 2.67 |
| 1-2 weeks | 9 | 2.53 | 0.17 | 2.80 | 1.36 | 3.00 |
| 2-4 weeks | 9 | 2.16 | 0.29 | 2.67 | 0.60 | 3.00 |
| 4-8 weeks | 13 | 2.45 | 0.16 | 2.67 | 1.10 | 2.90 |
| < 8 weeks | 19 | 2.09 | 0.13 | 2.13 | 1.20 | 3.00 |
| None* | 17 | 2.15 | 0.16 | 2.30 | 0.69 | 2.90 |
| ≤ 4 weeks* | 25 | 2.30 | 0.13 | 2.50 | 0.60 | 3.00 |
| > 4 weeks* | 32 | 2.24 | 0.10 | 2.39 | 1.10 | 3.00 |
| **Rehoming organization** |  |  |  |  |  |  |
| Laborbeaglehilfe* | 21 | 2.18 | 0.13 | 2.25 | 0.69 | 2.90 |
| Shelter Wermelskirchen * | 53 | 2.26 | 0.09 | 2.43 | 0.60 | 3.00 |
| **Residential area** |  |  |  |  |  |  |
| Suburb | 42 | 2.28 | 0.10 | 2.47 | 0.69 | 3.00 |
| Rural | 19 | 2.24 | 0.14 | 2.30 | 1.17 | 3.00 |
| City | 12 | 2.05 | 0.19 | 2.18 | 0.60 | 2.80 |
| Rural* | 19 | 2.24 | 0.14 | 2.30 | 1.17 | 3.00 |
| Urban* | 54 | 2.23 | 0.09 | 2.42 | 0.60 | 3.00 |
| **Family members** |  |  |  |  |  |  |
| ≤ 2 persons, no child | 19 | 2.14 | 0.16 | 2.30 | 0.60 | 2.90 |
| > 2 persons, no child | 13 | 1.85 | 0.17 | 1.67 | 0.69 | 2.80 |
| No child* | 32 | 2.02 | 0.12 | 2.06 | 0.60 | 2.90 |
| ≥ 1 child* | 33 | 2.41 | 0.09 | 2.67 | 1.06 | 3.00 |
| **Garden** |  |  |  |  |  |  |
| No | 11 | 2.03 | 0.24 | 2.24 | 0.60 | 3.00 |
| Yes | 53 | 2.21 | 0.08 | 2.40 | 0.69 | 3.00 |
| **Partner dog** |  |  |  |  |  |  |
| No (I1)* | 25 | 2.05 | 0.15 | 2.25 | 0.60 | 3.00 |
| Yes (I1)* | 49 | 2.33 | 0.08 | 2.44 | 1.20 | 3.00 |
| No (I2) | 22 | 2.07 | 0.16 | 2.27 | 0.69 | 3.00 |
| Yes (I2) | 52 | 2.31 | 0.08 | 2.44 | 0.60 | 3.00 |
| **Owner’s experience** |  |  |  |  |  |  |
| First dog* | 15 | 2.24 | 0.13 | 2.30 | 1.33 | 2.80 |
| Other dogs before* | 56 | 2.23 | 0.09 | 2.42 | 0.60 | 3.00 |
| **Dog classes** |  |  |  |  |  |  |
| No (I1) | 60 | 2.18 | 0.08 | 2.30 | 0.69 | 3.00 |
| Yes (I1) | 12 | 2.44 | 0.19 | 2.64 | 0.60 | 3.00 |
| No (I2) | 49 | 2.28 | 0.08 | 2.41 | 1.10 | 3.00 |
| Yes (I2) | 16 | 2.17 | 0.18 | 2.46 | 0.69 | 3.00 |
| **Obedience training** |  |  |  |  |  |  |
| No (I1) | 28 | 2.08 | 0.11 | 2.27 | 0.69 | 2.80 |
| Yes (I1) | 44 | 2.31 | 0.09 | 2.49 | 0.60 | 3.00 |
| No (I2) | 11 | 1.77 | 0.16 | 1.50 | 1.10 | 2.80 |
| Yes (I2) | 53 | 2.34 | 0.08 | 2.50 | 0.69 | 3.00 |
| **Punishment** |  |  |  |  |  |  |
| Frequently (I1) | 38 | 2.39 | 0.10 | 2.60 | 0.60 | 3.00 |
| Rarely (I1) | 31 | 2.07 | 0.11 | 2.24 | 0.69 | 2.93 |
| Frequent ly(I2) | 27 | 2.37 | 0.11 | 2.60 | 1.06 | 3.00 |
| Rarely (I2) | 37 | 2.19 | 0.10 | 2.25 | 0.69 | 3.00 |
| **Rewarding** |  |  |  |  |  |  |
| Frequently (I1) | 55 | 2.28 | 0.08 | 2.48 | 0.60 | 3.00 |
| Rarely (I1) | 16 | 2.08 | 0.15 | 2.22 | 1.10 | 2.93 |
| Frequent ly(I2) | 41 | 2.33 | 0.09 | 2.43 | 0.69 | 3.00 |
| Rarely (I2) | 24 | 2.11 | 0.13 | 2.33 | 1.10 | 2.93 |
